# An integrated instance segmentation and deep feature fusion model for robust leaf species classification in natural environments

**DOI:** 10.3389/fpls.2026.1893779

**Published:** 2026-10-15

**Authors:** K. Arunkumar, S. Leninisha

**Affiliations:** 1 Research Scholar, School of Computer Science & Engineering, Vellore Institute of Technology (VIT) Chennai, Kanchipuram, Tamil Nadu, India; 2 Faculty, School of Computer Science & Engineering, Vellore Institute of Technology (VIT) Chennai, Kanchipuram, Tamil Nadu, India

**Keywords:** deep feature fusion, leaf segmentation, mask R-CNN, precision agriculture, PSO optimization, species classification

## Abstract

**Introduction:**

The recognition of leaf segments and species in nature is a difficult process due to the changes in brightness, too much clutter in the background, and the overlapping of leaves due to which the accuracy falls down for 20-40% when using laboratory scans instead of field-ready applications.

**Methods:**

This research presents a unified framework of deep learning that includes Mask R-CNN -based instance segmentation and deep feature fusion in order to overcome this problem. The process of extracting a number of leaves took place through Detectron2 and the excessive stream extractor that included ResNet-50 and VGG-16 used in conjunction with other descriptive methods allowed providing a joint representation of 6,148 dimensions. The classifier was trained according to three methods: Baseline, PSO (particle swarm optimization), and Grid search.

**Results:**

The masked fusion system cut off the noise in the background while retaining the important botanical features with the help of achieving the mean Intersection-over-Union equal to 0.752, detection precision of 98.39%, and mAP@0.5 of 0.8291. The ResNet-50 model enhanced with PSO techniques has the best performance among other technologies based on ResNet-50 (90% accuracy, F1 score 0.9002), while the technology of Grid search revealed a little higher result for the special techniques such as ResNet-50 plus traditional features (F1 score = 0.9135) which means that the efficiency of the methodology is contingent to the type of the features not the fact if the technology is better than the other.

**Discussion:**

Comparative and ablation data analysis proved the efficiency of the instance segmentation used in combination with multi-mode fusion especially when used in occlusion-prone conditions. The system offers a scalable application for smart agriculture and biodiversity monitoring while being a good example of bridging the gap in automated environmental intelligence.

## 1 Introduction

The increasing need of food security, and biodiversity conservation in the world has triggered the creation of automated plant recognition systems (Mohanty et al., 2016; Pound et al., 2017; Ferentinos, 2018). The technologies are precursors to precision agriculture, ecological monitoring and botanical conservation (Li et al., 2021; Zong et al., 2025). While deep learning models achieve high accuracy on laboratory datasets like Flavia or Swedish Leaf, a performance gap of 20–40% persists when these models encounter unpredictable field conditions (Kumar et al., 2021; Lee et al., 2023; Van Hieu et al., 2023). This research will target this disparity via the use of a “Masked Fusion Design” allowing for pixel-mask processing so that feature extraction is confined to the leaf area itself, thus eliminating surrounding obscurants that include the ground (He et al., 2017), sky and shadows that generally obstruct global classifier functioning (Keivani et al., 2020; Dong et al., 2024; Xu et al., 2025).

The classical approaches to identification that rely on manual color methods such as GLCM (Gray-Level Co-occurrence Matrix) or Fourier descriptors encounter problems with natural occlusions (Pushpanathan et al., 2021; Picek et al., 2022; Khanna et al., 2024). On the other hand, the modern Convolutional Neural Networks (CNNs) usually unintentionally incorporate background noise in their feature vectors (Sahaya Sakila et al., 2025) (Aguerchi et al., 2024; Saini and Nagpal, 2024). This study presents a two-stream backbone that incorporates ResNet-50 and VGG-16 to examine the trade-offs among the macro-level venation pathways and the fine-grained texture granularity (Al-Gaashani et al., 2023; Sonali and Dhotre, 2024; Sireesha et al., 2025; Zhang et al., 2025). Though the combination of these architectures into a 6148-dimensional vector runs a risk of over-parameterization, this architecture measures their discriminative capability of combined embeddings in cases where they are fed by morphological descriptors (Sun et al., 2017; Pearline et al., 2019; Raghukumar and Narayanan, 2020).

To offer strength, the study sets to offer comparative analysis of optimization techniques. This paper looks at how beneficial a metaheuristic solution, such as Particle Swarm Optimization (PSO), is when competing against a systematic method, such as Grid Search, at optimizing high-dimensional fused feature vectors. In the former case, it has been noted that the latter gives better results in cases where multiple features are present (Islam et al., 2019; Bedi and Gole, 2021). On a custom 392 image dataset of 10 Mediterranean trees species in uncontrolled conditions in Chennai, India, performance is confirmed (Wäldchen and Mäder, 2018; Joly et al., 2024; Franceschi et al., 2025; Moustakas, 2025). This set is the one that compares model generalization with inter-leaf occlusions and/or motion under the wind (Triki et al., 2021; Wang et al., 2024). This paper unites the Mask R-CNN segmentation with automated hyperparameter optimization to form a powerful procedure of precision identification of plants in the field (Liu et al., 2018; Hossain et al., 2019; Qadri et al., 2024).

Recent breakthroughs in deep learning technology have made it easier to analyze agricultural images by using convolutional neural networks, attention mechanisms, and hybrid feature learning strategies. A thorough systematic review notes the growing importance of using deep learning architectures in agriculture as it relates to efficiency, explainability, and reproducibility of models (Rasul, 2026). More recent studies on identifying plant diseases and medicinal plants have also shown that joining different visual features and using lightweight deep learning technologies lead to better classification results and improved use of these technologies in practice (Tran et al., 2026; Wang et al., 2026),.

Feature fusion has emerged as a successful strategy for improving prediction results in precision agriculture. The combination of multiple sensory feature fusion with attention-based learning has been successfully employed in high-throughput phenotyping of crops, thereby improving the accuracy of the estimation of agronomic characteristics (Fan et al., 2025). In the same way, combining geometric information with deep visual features has improved object detection and recognition ability of autonomous agricultural systems (Singh et al., 2026). Based on the above developments, the proposed framework uses the combination of deep convolutional features with the crafted set of morphological features for accurate and effective leaf species classification in uncontrolled outdoor imaging conditions.

The main objective of this study will be to come up with a framework field-ready, which will be precise to non-controlled environment. The precise objectives will be: Train a Mask R-CNN with a Masked Fusion Design in order to make sure that feature extraction is not sensitive to environmental noise. Explore the trade-offs between deep structural encodings (ResNet-50/VGG-16) and conventional morphological feature sets. Assess how effective the metaheuristic (PSO) and systematic (Grid Search) processes are in adjusting classifiers. Study resistance to the impacts of real life such as movement aroused by wind and the overlay of leaves using an exclusive dataset with vegetation typical for the Mediterranean flora.

Leaf classification is crucial for reliable identification of a plant species within a natural habitat, thus paving the way for efficient and intelligent phytoprotection systems. Accurate identification of the species will help in the early detection of invasive species while contributing towards biodiversity documentation and ecosystem health monitoring. In agricultural conditions, automated leaf classification can bring about improvements in crop management by contributing towards species inventory, vegetation monitoring, and timely field assessments, while freeing farmers from the labor-intensive surveys. In view of the fact that the proposed system combines instance segmentation with feature extraction techniques allowing it to work even under occlusion, different backgrounds, and in case of poor illumination, it can be instrumental in executing the tasks of plant identification in the field that are of great importance to phytoprotection and ecological monitoring systems.

### 1.1 Research novelty

Both Mask R-CNN segmentation and CNN-based feature extraction have already previously been used for the purpose of plant and leaf recognition, but most of the work in this area sees segmentation as a preprocessing task or uses only one deep learning architecture and backbone in order to extract features from images. As for the new approach suggested by the authors of the current study, it implements a segmentation-guided masking feature fusion strategy, which means that the masks created by Mask R-CNN at pixel level help to extract features. Unlike traditional feature concatenation, the implementation of the suggested fusion makes it possible to merge structural features provided by ResNet-50, fine-grain textural features derived from VGG-16 and morphological features created manually in one representation that retains proper biological information and minimizes the effect of environmental noise. It should also be noted that the current research makes a thorough comparison between the methods of Grid search and PSO in the same conditions for feature representations proving that the efficacy of optimization does not depend on the method used, but rather on the characteristics of the feature representation chosen.

### 1.2 Key contributions

- The proposed framework applies segmentation-based classification methods to facilitate extracting consistent features from the leaf region affected by environmental noise due to uncontrolled outdoor conditions.
- A representation of features with 6148 dimensions has been created based on hybrid characteristics composed of deep features from two famous models, ResNet-50 and VGG-16, and four features obtained by one of the morphological methods so that extensive but understandable features could be used at the same time.
- A series of experiments aimed at analyzing various hyperparameter optimization techniques have been carried out, which confirmed the fact that Particle Swarm Optimization (PSO) converges faster while Grid Search is more stable in case of using selected feature sets.
- The framework was tested on a custom dataset that included ten plant species of Mediterranean origin collected from nature in Chennai, India, as well as additional testing on the well-known Flavia dataset to obtain the proof of generalization potential of the approach.

The rest of this paper is organized in the following way: Section II describes the provided custom dataset, the Mask R-CNN segmentation, and the mathematical model of the feature fusion and optimization. In section III, there is a report on experimental findings, such as segmentation scores (mIoU 0.752) and classification scores. Section IV presents the conclusion of the research, namely addressing the sensitivity of boundaries in serrated margins and trade-offs of the dual-backbone solution.

## 2 Methodology

The proposed methodology consists of instance-level segmentation, multi-stream feature extraction, and evolutionary optimization for the purpose of carrying out reliable leaf species classification in an outdoor environment. The pipeline starts off with the segmentation based on Mask R-CNN for separating single leaves against complex backgrounds that involve overlapping foliage and shadows and varied illumination (Wagle et al., 2021; Haridasan et al., 2023; Kavitha et al., 2023). The pre-processing of segmented leaf areas is done by cropping the leaf area to remove the background noise (Gautam et al., 2025) (Ma et al., 2023; Tomar et al., 2025). The ResNet-50 and VGG-16 networks are used to derive deep feature maps, and morphological descriptors are used to elicit the crucial structural attributes of leaves (Nayak and Raghatate, 2024; Sherly and Sonia, 2024). These complementary characteristics are joined together in a single representation and into a fully connected classifier (Chowdhury et al., 2024). Hyperparameters are optimized by training with three training configurations to enhance the model performance and stability, which are baseline, grid search, and Particle Swarm Optimization (PSO). Evaluation of the system is based on the standard measures such as the IoU, mAP, accuracy, precision, recall, and F1-score.

### 2.1 System overview

As shown in **Figure 1**, the suggested architectural structure aims to achieve accurate leaf species identification under environmental conditions, with the presence of clutter, leaf overlapping, partial leaf occlusion, and variability in illumination level (Wu et al., 2021; Yao et al., 2024). The architecture has three main components: instance separation, multi-stream attributes request using masking fusion technique, and classification with hyper parameter optimization. At the beginning, Mask R-CNN technology is used to implement the instance-level separation of single leaves from the input image of high resolution (512 × 512 × 3) (Saleem et al., 2019; Tiwari et al., 2022; Uddin et al., 2023). The pixel-level masks and bounding boxes generated by the segmentation network allow for accurate localization of every leaf while eliminating interference from other plants as well as background objects. The showpiece comes out as the segmented region of interest is cut out of the visible area through the masked leaf image (128 × 128 × 3) to assist in sending only relevant leaf pixels to the feature extraction process (Zhao et al., 2015; Biurrun et al., 2025). The resultant leaf image is fed into two different AI techniques to extract features (Priya, 2021). While the first branch uses the ResNet-50 network trained previously to acquire high-level patterns of shapes, veins, and textures, the output is one 7 × 7 × 2048 feature map (Kumar et al., 2021; Kumar et al., 2023). At the same time, the second branch uses the VGG-16 model trained earlier to again save information on shapes and edges, producing one 7 × 7 × 512 feature map (Zaki et al., 2020; Kaur et al., 2022; Puranik et al., 2024). Finally, some features from the morphological description, including shape and Hu signature, are extracted to gain additional information on structure that may not have been fully captured through the deep convolutional models.

**Figure 1:**
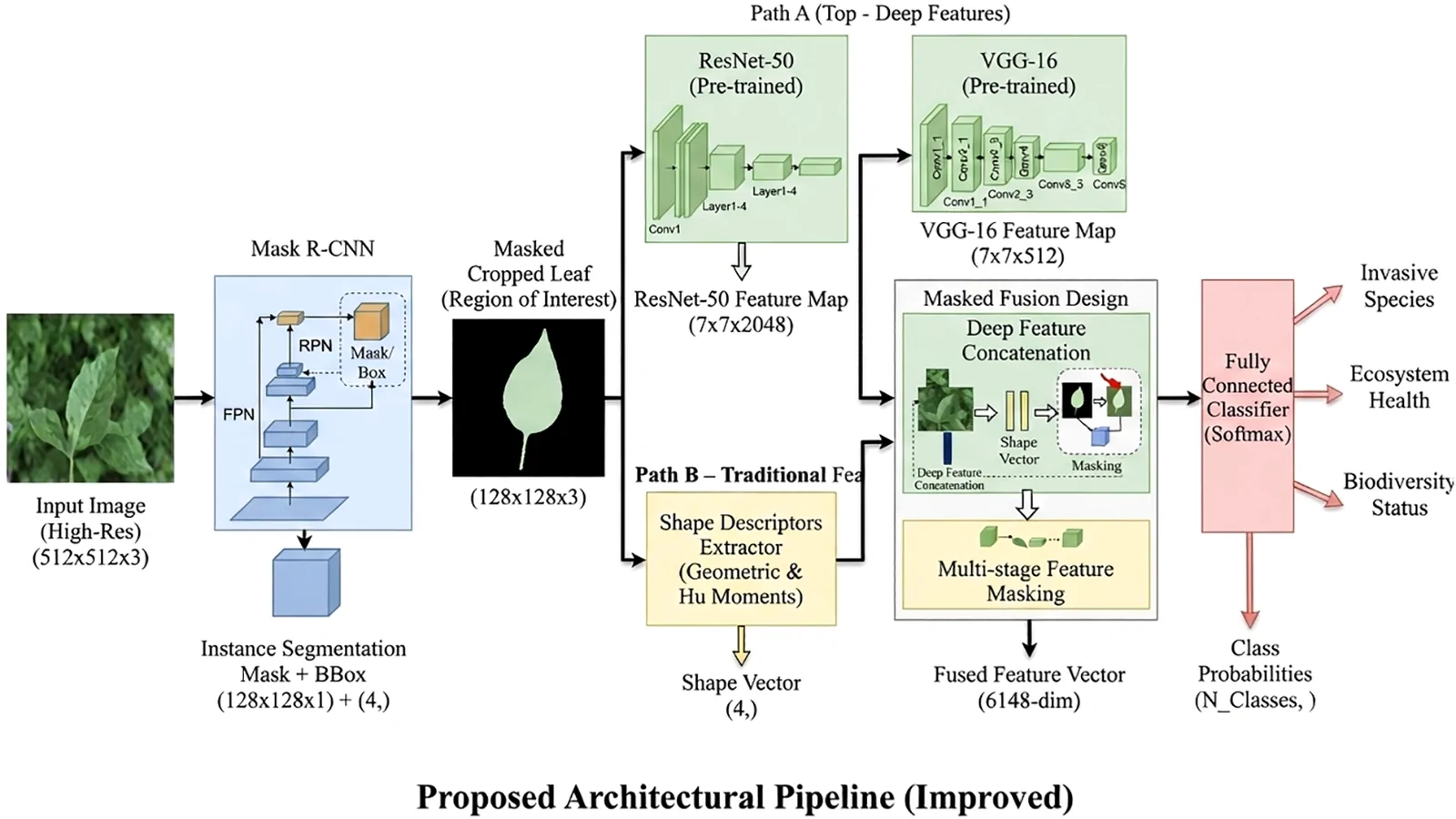
Overall architecture of the proposed instance segmentation and deep feature fusion framework for leaf species classification.

Then, the created handcrafted and deep characteristics are combined using the proposed Masked Fusion Design. In the method, the deep features are concatenated and then masked using multi-step feature masking, where a pixel-wise segmentation mask is applied to eliminate any remaining background signal from the fusion of the features. The end result is a combined 6148-dimensional fused feature vector which combines both the semantic, textural, and geometrical representation of the leaves and allows the crucial parts of the leaf to be recognized and the irrelevant ones to be omitted. The fused feature vector is then fed into a fully connected classifier with a Softmax layer for classification of the leaf species. The training of the classification relied on the use of three different optimization techniques known as Baseline, Grid Search, and PSO. In order to avoid overfitting and ensure that the best validation performance is obtained, the early stopping method has been successfully employed during the training. The framework that has been developed proved to be effective through achieving stability of convergence and good classification results.

Targeted Environmental Robustness: Mention that the modular pipeline is specifically engineered to counteract a typical 20–40% drop in accuracy when moving from controlled to natural settings by isolating “biological meaningful pixels” (Qiu et al., 2021; Malik et al., 2022; Thanikkal et al., 2023).

Stage-Wise Contribution Explains that the initial stage (Mask R-CNN) is not only an object detector but a sort of spatial filter to get rid of all background noise (soil, shadows, overlapping plants, etc.) and no feature encoding happens (Pujari et al., 2016; Dai et al., 2024; Meraj et al., 2024).

### 2.2 Data acquisition and annotation

To guarantee that the collection of samples occurred in a controlled and natural environment while also ensuring the capture of a variety of biological samples, the data was gathered outdoors. In the image-acquiring process, a Canon EOS 80D DSLR camera was used. An EF-S 18-55mm f/3.5-5.6 IS STM lens was fitted to the camera, which was held in place at a distance of around 20–30 cm from the surface of the leaf. The lighting conditions during the acquisition of the images were not controlled but varied based on natural daylight, cloudy skies, etc. Furthermore, they aimed to establish a protocol whereby samples will be captured from various positions and angles. Once the image-capturing stage was accomplished, the samples were labeled using a computer program called LabelMe.

### 2.3 Description of the dataset and environmental context

This research article provides data from 392 pictures of plants belonging to ten types of tree species found in the Mediterranean region. Despite the fact that the species under examination are either native to or have a strong link with the Mediterranean ecosystem, the pictures were taken from registered plants found in the area of Chennai in India. As a result, the analyzed species have peculiar features that correspond to the environmental characteristics of a particular area, rather than those of the Mediterranean region. There are several reasons which may have contributed to the distinctive morphology of leaves in the region, including temperatures and rainfall, soil composition, and other variants of cultivation. Therefore, though the idea proposed in the paper can be applied to the area where the data were gathered, its results need validation in a wider context. The species analyzed in the paper include Hedera helix, Morus alba, Viburnum tinus, Corylus avellana, Quercus ilex, Laurus nobilis, Pittosporum tobira, Crataegus monogyna, Cercis siliquastrum, and Acer campestre. The data are summarized in **Table 1**. The model was evaluated using stratified 5-fold cross-validation instead of simply splitting the sample into testing and training groups.

**Table 1:** Summary of the proposed leaf species dataset used for model development and evaluation.

Species	Total images
Hedera helix L.	52
Morus alba L.	36
Viburnum tinus L.	47
Corylus avellana L.	36
Quercus ilex L.	38
Laurus nobilis L.	37
Pittosporum tobira	38
Crataegus monogyna Jacq.	37
Cercis siliquastrum L.	39
Acer campestre L.	32
Total	392

#### 2.3.1 Environmental generalization

To support the “Field-Ready” claim of the proposed framework, the dataset was specifically curated to include challenging environmental variables often absent in standardized, static laboratory datasets. In **Figure 2**, the dataset’s sample data shows precisely the type of complexity that the model will be dealing with in practice. To mention a few, the dataset includes the following:

**Figure 2:**
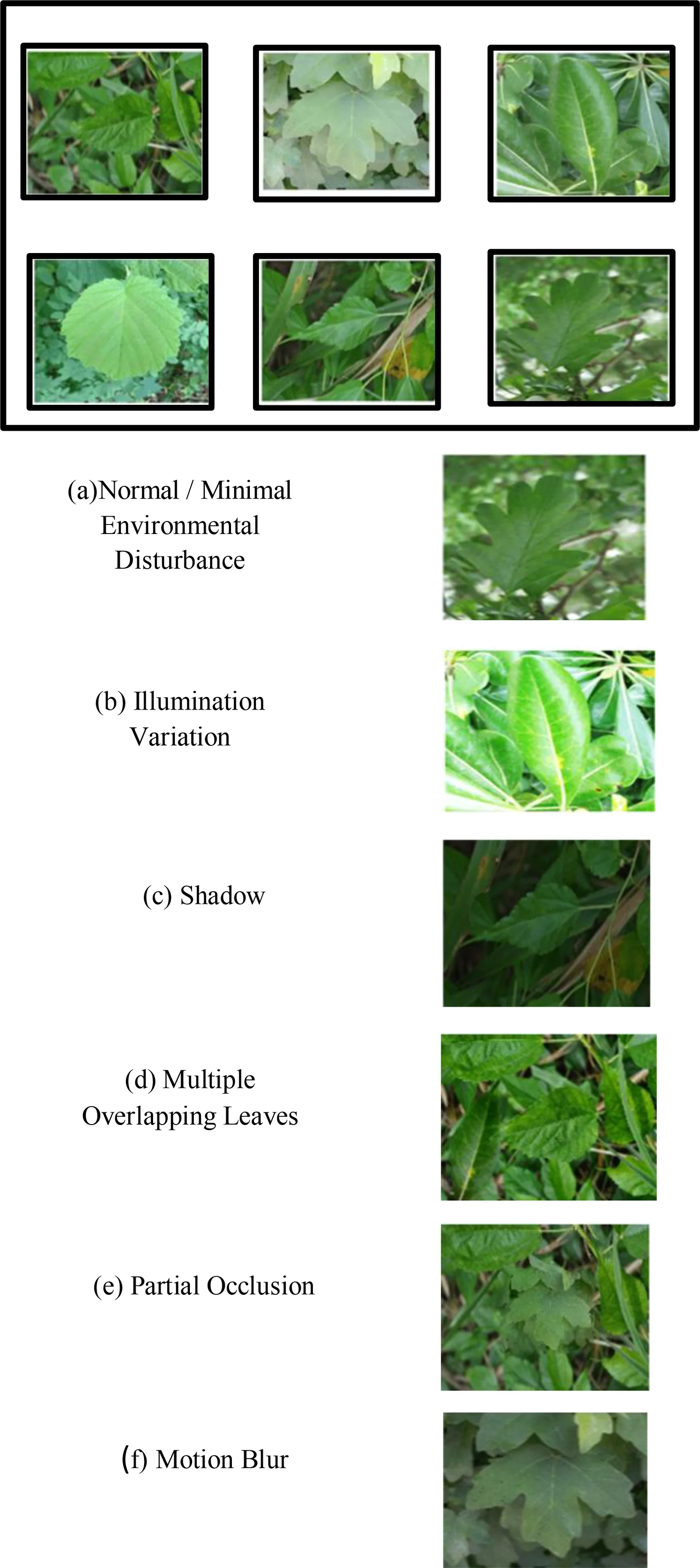
Representative samples from the annotated leaf image dataset collected under natural outdoor environmental conditions. **(a)** Normal / Minimal Environmental Disturbance, **(b)** Illumination Variation, **(c)** Shadow, **(d)** Multiple Overlapping Leaves, **(e)** Partial Occlusion, **(f)** Motion Blur.

Wind-Generated Blurred Photograph: Get a picture of the leaves in motion and check how effectively the Mask R-CNN that is capable of segmenting images manages to depict the edges of the picture clearly (Chanyal et al., 2022; Valdez et al., 2022).

Layers of Leaves Overlapping: Pictures that include about 1–5 leaf instances, which can mix their parts to produce the proper canopy density.

Bright and Dark: A contrast between deepest shadows and strongest sun rays, and also between the tree way and the certain details in the soil, tree roots and the perspective into the ground surface.

This focus on environmental complexity, visually evidenced in **Figure 2**, ensures the model is evaluated against real-world “noise,” providing a more rigorous test of generalization than traditional single-leaf scans.

#### 2.3.2 Data statistics and annotation

There are a total of 392 leaf images in the data which has been labelled, and these leaf images represent 10 different Mediterranean tree species. Five-fold cross validation stratified was used for evaluating the model so that the proportion of the class of each species is consistent across all the folds. During the five iterations of the cross validation process, nearly 80% of the data was used for model building, while 20% was kept aside for independent test dataset. Segmentation process used ground truth manually created using LabelMe version 5.2.0, which helps in providing pixel-level resolution for segmenting masked images.

The species labels which were given were done using proper botanical recognition references prior to the data collection so that there is concordance between the taxonomical identity of any specimen and its segmentation mask.

##### Dataset size and limitations

While the proposed dataset includes difficult outdoor factors such as occlusions, background clutter and changes in lighting, its size (392 images across 10 species) is not enough for deep learning training. To prevent overfitting various regularization methods were applied, including transfer learning with ImageNet pretrained networks, dropout technique, early stopping, stratified dataset partitioning and comparative hyperparameter optimization. At the same time, the number of samples per species remains limited which makes it impossible for the model to process full intra-class variability in different conditions. Thus, the results should be taken into account after the context of the current dataset.

### 2.4 Evaluation metrics and averaging strategy

Performance of the 21 configurations is evaluated using the three metrics of Accuracy, Precision, and Recall, as well as F1-score. For a comprehensive assessment of global performance, technology specifications are calculated through Micro-averaging. Micro-averaging aggregates true positive, false positive, and false negative data globally in all the classes. Hence, all reported values take into account the overall classification success rate in the situation. As a result of this, values of Accuracy and Recall turn out to be the same because they derive from the same overall number of successes relative to the global pool of instances which were tested.

### 2.5 Pre-processing and masked fusion design

Before any preprocessing or feature extraction was conducted, all of the available data was split into three main sets; namely, training, validation, and test sets. All three sets were divided based on stratified random sampling so that species’ class distributions could be preserved among the observed leaf species. The training and validation sets were used to develop and fine-tune the expected model while keeping the test set exclusively for performance evaluation purposes. The analysis ensured that all experiments could be reproduced by using a fixed random seed during the dataset partitioning and model initialization stage. The dataset partitioning was performed prior to preprocessing so that no information from the validation and test sets was introduced into the training stage since this could lead to data leakage. After the partitioning process had been completed, the necessary preprocessing steps (i.e. the instance segmentation with the Mask R-CNN technology) were applied identically to each split dataset.

In connection with data augmentation, it should be noted that synthetic data generation methods like rotating the photos in a random manner, performing shots and flips, scaling or using color jitter are being replaced by natural variability, which was found in the gathered dataset. Thus, illumination, viewing angle, occlusion and background complexity already provide sufficient variety of input for the training of the model and make its learning resilient to real-world shifts without making any use of synthetic data. In addition, the large-scale data set prevents from overfitting and ensures that the training samples represent characteristics of the target domain sufficiently well.

Data pre-processing is important to ensure the data is consistent and improves the performance of the model. This is especially true for images captured in the wild, where factors like lighting, occlusion, and scale create a great variation of the data. In a laboratory setting, the circumstances of data capture can be regulated, but achieving leaf images in the wild requires a consistent approach for adapting the data input and distinguishing important anatomical features. The architecture of the model defined based on the pre-processed data consists of a few branches for the features. The use of more sophisticated methods of merging the features such as those based on attention principles, adaptive gating, and transformer-based merging allows the system to adaptively change scores of the features. However, the proposed architecture does not use any of these technologies; instead, it relies on rather straightforward concatenation of the features. Concatenation may not be a very sophisticated technique, but it is a simple process, which makes it possible to retain the distinctive features coming from the ResNet, VGG, and shape-based majors without raising risk of overfitting, which may be caused by too complex methods of processing. Furthermore, empirical studies showed that the proposed concatenation gives satisfactory results, thus, there is no sense to use more layers for fusing.

#### 2.5.1 Dimensionality standardization and normalization

All the images are scaled to a constant resolution that is supported by both the Mask R-CNN and the dual-stream CNN backbones (Islam et al., 2020). This standardization guarantees the homogeneous input dimensions across the entire pipeline of deep learning and the reduction of its computational cost, as well as optimization of resources on the GPU (Graphics Processing Unit). After resizing, normalization of the pixel intensity is carried out to eliminate the illumination induced variance. Normalization of a raw image $I_{raw}$ is given by Equation 1:

(1) $$I_{norm}=\frac{I_{raw}-\mu }{\sigma }$$

Where μ and σ are the mean and the standard deviation respectively. Normalization Alignment in this system is done against ImageNet distribution. This guarantees a consistent gradient propagation and faster convergence by matching the distributions of the data obtained by the field with the pre-trained weights of the encoders of the ResNet-50 and VGG-16 encoders.

#### 2.5.2 Masked fusion design: pixel-level gating

To bridge the laboratory-to-field performance gap, this study introduces a Masked Fusion Design. Unlike standard global classifiers that process the entire image, this design utilizes a Pixel-Level Gating mechanism. Once initial segmentation results are obtained, a binary mask M(x,y)is generated for each instance to isolate the target leaf pixels given by Equation 2:

(2) $$I_{masked}(x,y)=I_{raw}(x,y)\cdot M(x,y)$$

This procedure acts as a spatial filter, ensuring that the subsequent feature encoding is strictly restricted to the leaf surface. By implementing this mask-guided cropping using extracted bounding box coordinates $\left(x_{min},y_{min},x_{max},y_{max}\right)$, the system achieves the Elimination of Environmental Confounders (Gowthami et al., 2021; Sedimo et al., 2025; Umamaheswari, 2025).

This masking procedure is crucial for obtaining accurate results since it stops deep learning techniques from acquiring knowledge about non-biodiversity characteristics like soil structure, tree branch structure, or sky color. Thus, the model is able to concentrate on the properties of the leaf such as its veins and the structure of its edges, which increases its ability to distinguish between different plant species even in the presence of obstacles in environmental conditions.

### 2.6 Hyperparameter optimization

In order to obtain trustworthy performance and reaching convergence with the model, hyperparameter tuning was thoroughly conducted based on the validation set. The necessary parameters were defined via empirical sensitivity analysis where the parameters were modified consecutively depending on their impact on validation loss and accuracy. Therefore, the final model setup represented the balance between convergence and globalization, and the model was trained without experiencing overlearning. The parameters used in the process are outlined in **Table 2**.

**Table 2:** Optimal hyperparameters and training configuration.

Parameter	Selected value
Learning Rate	0.001
Batch Size	32
Epochs	50
Population Size	30
Maximum Iterations	100
Mutation Probability	0.2
Optimizer	Adam

### 2.7 Mask R-CNN-based leaf segmentation

The adoption of mask R-CNN for the instance-level extraction of leaves is warranted by the better capacity of this technique in object detection and in clearly defining the object border in a complex natural environment (Demilie, 2024). In contrast to global image segmentation methods, Mask R-CNN offers pixel-based masks with precision when there is an occlusion or an aggregation of leaves as well as the background object which are common in the real-world agricultural ecosystems. The end-to-end segmentation process is shown in **Figure 3** and starts with the generation of region proposals and ends with instance-specific pixel masks.

**Figure 3:**
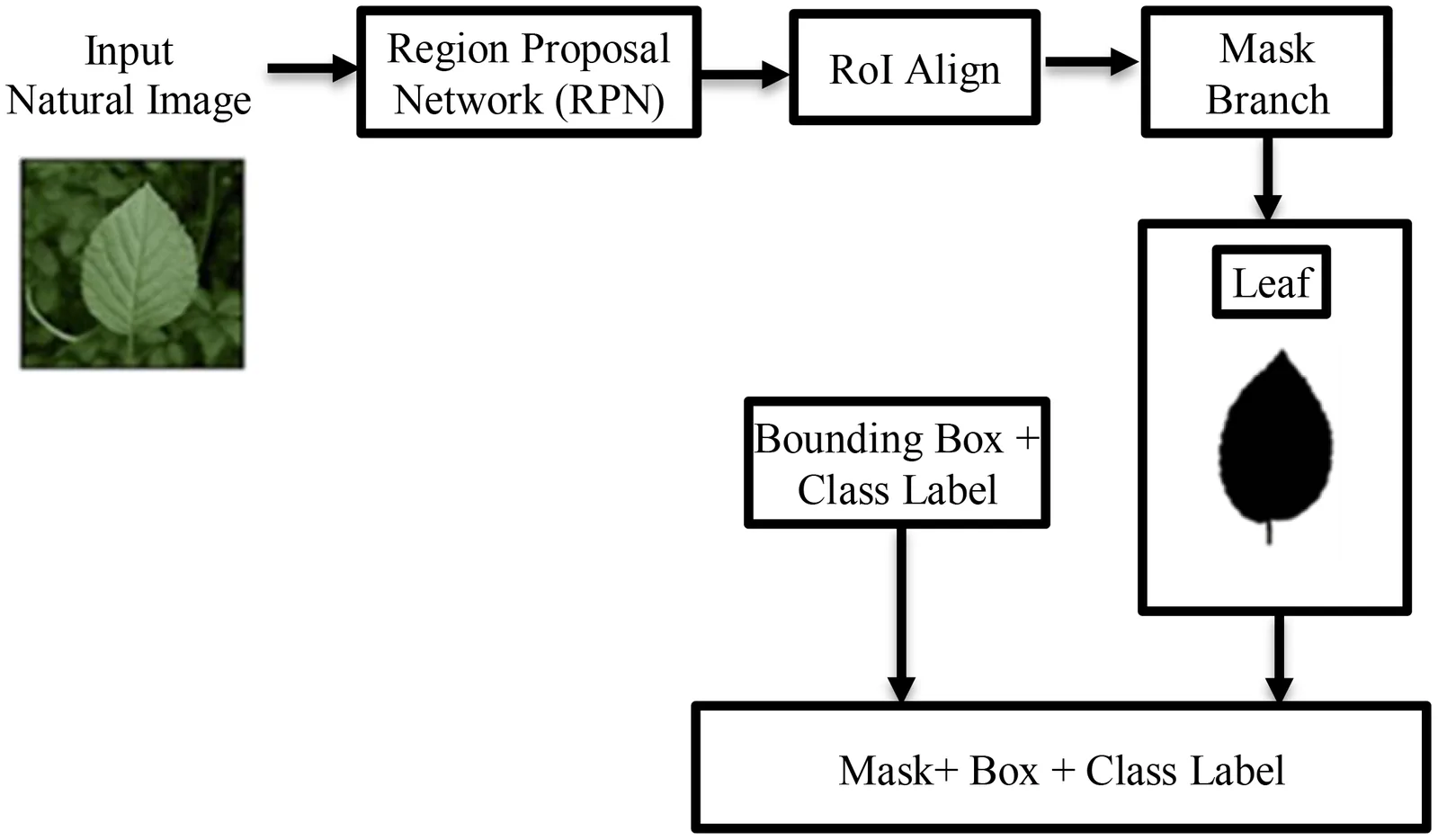
Workflow of the proposed Mask R-CNN-based instance segmentation process for precise leaf extraction.

The structure of the model is built upon the Mask R-CNN framework, where the description of the standard Faster R-CNN classifier has been improved with addition of the second branch that predicts pixel-wise masks. The Region Proposal Network (RPN) is the one that is generating proposals for the classes, while RoIAlign achieves spatial relation retention, making it possible to extract high-quality features. The network generates both bounding boxes of the objects and the exact forms of their leaves that will be required for feature computation. As for the Mask R-CNN, it optimizes multi-task loss function allowing the scheme to learn all the three objectives together. The total loss is defined as follows (Equation 3):

(3) $$L=L_{cls}+L_{box}+L_{mask}$$

where $L_{cls}$ is the classification loss, $L_{box}$ represents the Smooth-L1 regression loss for bounding box refinement, and $L_{mask}$ denotes the pixel-wise binary cross-entropy loss (Keivani et al., 2020). This joint optimization ensures precise identification and shape-level segmentation, which is critical for capturing the morphological diversity across leaf species.

### 2.8 Mathematical formulation of the fusion strategy

The proposed architecture integrates features from three distinct branches: a ResNet-based deep feature branch, a VGG-based deep feature branch, and a shape-based descriptor branch. Let ${\text{f}}_{R}\in {\mathbb{R}}^{n}$, ${\text{f}}_{V}\in {\mathbb{R}}^{m}$, and ${\text{f}}_{S}\in {\mathbb{R}}^{p}$ denote the feature vectors extracted from the ResNet, VGG, and shape-based branches, respectively.

To create a unified representation, these feature vectors are integrated via concatenation (Equation 4):

(4) $$\text{F}=[{\text{f}}_{R}⊕{\text{f}}_{V}⊕{\text{f}}_{S}]$$

where $\text{F}\in {\mathbb{R}}^{\left(n+m+p\right)}$ represents the concatenated feature vector. This fused representation is then projected into the target label space through a fully connected layer (Equation 5):

(5) Z=WF+b

where $\text{W}\in {\mathbb{R}}^{K\times (n+m+p)}$ represents the weight matrix, $\text{b}\in {\mathbb{R}}^{K}$ denotes the bias vector, and K corresponds to the total number of leaf species. The final class probability distribution is obtained via the softmax function, where the probability for the i-th class is calculated as (Equation 6):

(6) $${\hat{y}}_{i}=\frac{e^{z_{i}}}{\sum_{j=1}^{K}e^{z_{j}}}$$

Through the method of direct fusion, one can keep the identity of its different characteristics, as it provides an efficient integration method that reduces challenges posed by other complex methods of gating regarding overfitting.

### 2.9 Feature extraction

Once the segmentation process is complete, high-order discriminative features are extracted from individual leaf regions so that both in-depth textural-venation information and natural morphologic characteristics are captured (Chaudhury and Barron, 2018; Banzi and Abayo, 2021). In this direction, multi-channel representation of deep convolutional features and crafted shape reflectors is used (Agarwal and Venkat, 2020). This mixed system (**Figure 4**) uses the hierarchical abstraction ability of deep neural networks and retains biologically explainable geometrical information that is pertinent to the differentiation of species.

**Figure 4:**
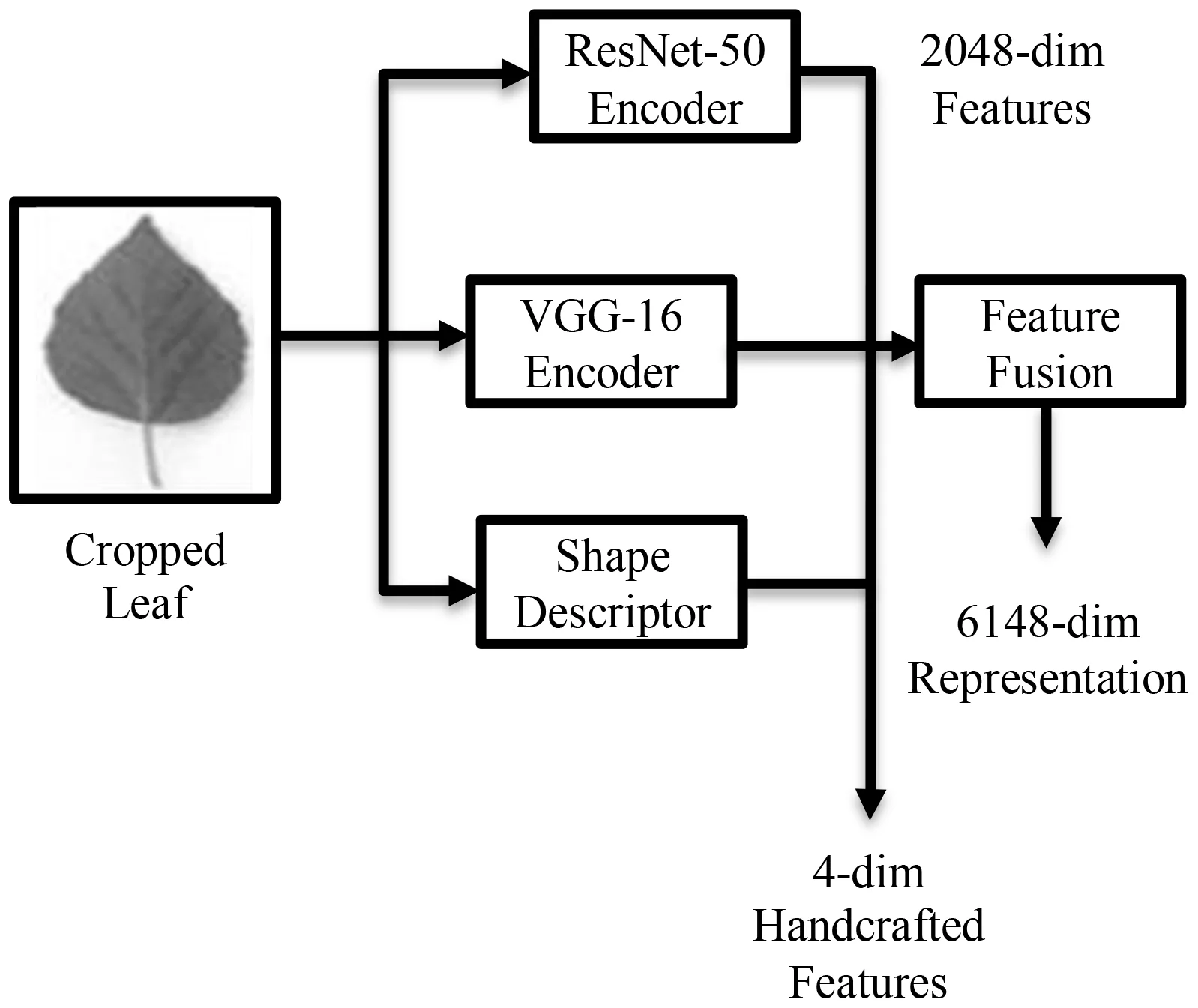
Multi-stream feature extraction framework integrating deep convolutional and morphological descriptors for leaf species classification.

#### 2.9.1 ResNet-50 feature encoder

ResNet-50, a residual CNN architecture pretrained on ImageNet, is employed due to its well-established capacity for learning multi-scale texture and venation patterns, which are critical for leaf classification. Formally, given an input cropped leaf region L, the deep representation F_res_ is given as Equation 7:

(7) $$F_{\text{res }}=\phi_{\text{res }}(L)$$

Where $\;\phi_{\text{res }}\text{(·)}$ denotes the ResNet-50 forward inference function. The residual learning framework follows Equation 8:

(8) $$y=F(x,W_{i})+x$$

With F(·) representing stacked convolutional transformations and shortcut identity mapping ensuring efficient gradient propagation. The output tensor is globally averaged to yield a 2048-dimensional descriptor.

#### 2.9.2 VGG-16 feature encoder

Complementary to ResNet-50, VGG-16 is exploited for its ability to preserve fine-scale edge information and venation continuity. VGG-16 employs sequential stacked convolutional kernels of size (3 * 3), operating as Equation 9:

(9) $$F_{\text{vgg }}=\phi_{\text{vgg }}(L)$$

Resulting in a 4096-dimensional activation after the fully-connected layers. The distinct structural properties of VGG filters augment the ResNet representation by emphasizing hierarchical, fine-grained texture features.

#### 2.9.3 Traditional shape feature extraction

In order to facilitate morphological interpretation and to extract the natural structure, custom shape descriptors are pulled from the selected leaf portion. The binary segmentation mask is defined by M (x,y) where a value of 1 denotes the leaf portion while 0 denotes the background.

The area of leaf is calculated as in Equation 10.

(10) $$\text{Area}=\underset{\left(x,y\right)}{\sum }M(x,y)$$

where M(x,y)is the binary mask value at pixel location (x,y).

The leaf perimeter is calculated as Equation 11

(11) $$\text{Perimeter}=\underset{\left(x,y\right)}{\sum }∥\nabla M(x,y)∥$$

where ∇M(x,y)denotes the gradient magnitude of the binary mask, representing the boundary pixels of the segmented leaf.

Leaf circularity is determined using Equation 12

(12) $$\text{Circularity}=\frac{4\pi \times \text{Area}}{{\text{Perimeter}}^{2}}$$

where Area and Perimeter are obtained from Equations 10 and 11, respectively. A higher circularity value indicates a shape closer to a perfect circle.

Leaf eccentricity is computed as Equation 13

(13) $$\text{Eccentricity}=\frac{\sqrt{a^{2}-b^{2}}}{a}$$

In the scenario in which the variables a and b are the semi-major axis and the semi-minor axis of the equivalent ellipse, the eccentricity value close to zero suggests a rounder shape while a value nearer to one indicates more elongated shapes of leaves.

The use of a four-dimensional shape descriptor that includes area, perimeter, circularity, and eccentricity makes it possible to obtain extra geometric information that helps improve the accuracy of deep feature description for identification of different leaf types.

The handcrafted feature set is limited to four geometric characteristics only, namely area, perimeter, circularity, and eccentricity, in order not to compromise the trade-off between morphological discrimination and computational efficiency. The presented three are the geometric characteristics selected for the current research as they reveal different shape properties of a leaf structure: dimension (area), outline (perimeter), constancy/compactness (circularity), and elongation (eccentricity). The selected four shapes are believed to provide enough information for distinguishing between leaf species and keep the features compact at the same time. The authors avoided using more complex geometric descriptors such as Hu moments, Fourier descriptors, or Zernike moments, since the main discriminative power of the authors’ methodology relies on deep features provided by ResNet and VGG models. Thus, the goal of using handcrafted descriptors is not their replacement but the addition of initial interpretable shape information. Limiting the handcrafted features to four descriptors contributes to reducing redundancy, shortening computation time and eliminating the problem of overfitting due to not very big size of the dataset.

### 2.10 Feature fusion strategy and dimensionality analysis

The feature fusion process is designed as a comparative investigation to evaluate the trade-offs between semantic depth and computational stability. Rather than assuming that increasing the feature space inherently improves accuracy, this study systematically tests the integration of multi-scale deep embeddings with geometric priors (Hussein et al., 2021).

The fusion method being presented is different from the traditional concatenation of features that are acquired from the entire image. Here, the process of extracting features is done only from the biologically significant pixels of the leaves obtained using Mask R-CNN masks. This ensures that every backbone obtaining features from the same leaf gets exclusive information coming from the leaf without the influence of nearby plants and background. For instance, ResNet-50 gets mostly the information about the vegetation and the organization of the leaves, while VGG-16 focuses more on texture and edges. The introduction of descriptors takes care of the preservation of some geometry features that are usually hard for CNNs to capture.

#### 2.10.1 Concatenation-based early fusion

The primary fusion strategy employs an early-stage concatenation layer. Let $V_{Res}$be the 2048-dimensional vector from ResNet-50, $V_{VGG}$be the 4096-dimensional vector from VGG-16, and $V_{Morph}$be the 4-dimensional morphological descriptor. The resulting fused representation $V_{Fused}$is defined as Equation 14:

(14) $$V_{Fused}=[V_{Res}⊕V_{VGG}⊕V_{Morph}]$$

This results in a 6148-dimensional vector aimed at capturing both high-level residual mappings and fine-grained textural granularity.

#### 2.10.2 Dimensionality and over-parameterization risk

The assessment of dimensionality against sample size is a vital part of this methodology. Although the 6148-D maximizes the potential semantic depth of the features of the leaves, it takes a great risk of over-parameterization, especially when considering the inherent limitations of the 392-image data (Pal and Kumar, 2023; Sharma et al., 2024).

This part directly verifies the hypothesis that multidimensional vectors can cause a sparse feature space, which can cause the “Curse of Dimensionality.” The methodology determines the best point at which the richness of features exceeds the danger of overfitting by comparing the performance of the 2052-D (ResNet + Traditional) configuration with the performance of the 6148-D (All Features) configuration. The resultant model obtained is not optimal because it reflects empirical stability, and not because it reflects the maximum feature concatenation.

Even though the fused representation contains 6148 features, it is not suggested that this high-dimensionality will lead to better classification results. To determine a good balance between richness and generalization, combinations of features were tested in experiments. The results show that simple feature representations like ResNet-50 coupled with morphological features perform as well or better than the complete fused representation. It should be noted that effective feature selection is more significant than increasing feature dimensionality.

The filtered feature combination selection is based on comparative analysis of many different combinations and thereby, the most suitable feature representation is selected based on generalization performance and discriminative ability. The chosen fused feature vector is then utilized in the classification phase in which a fully connected neural network learns the nonlinear decision boundaries required for distinguishing among the ten types of leaves. This approach guarantees that the classification model will be functioning in the best suitable feature space which results in a higher accuracy level.

### 2.11 Classification model

A fully connected classifier receives the optimized feature representation that is acquired from the feature fusion step to achieve a final classification of the leaf species. This classifier consists of three dense neural network layers where ReLU activation layers are used after the first two dense layers. A dropout procedure with a rate of 0.5 is applied to avoid overfitting. The training process uses the strategy of minimizing categorical cross-entropy loss with the Adam optimizer.

**Table 3** gives complete details about the configuration of the classifier in terms of the dimension of the input, number of neurons of each layer, and dropout parameters. This configuration is proposed to achieve a good balance for trade-off between the complexity of the model and its ability to generalize, eventually allowing the classifier to provide the most efficient posterior probabilities for the class during classification.

**Table 3:** Architecture and hyperparameter configuration of the proposed fully connected classifier.

Layer	Description
Input	6148-dim feature vector
FC Layer 1	6148 → 512 neurons, ReLU activation
Dropout	0.5
FC Layer 2	512 → 256 neurons, ReLU activation
Dropout	0.5
Output Layer	256 → 10 classes, Softmax
Optimizer	Adam
Learning Rate	0.001 (tuned in Grid/PSO)
Max Epochs	500
Early Stopping	50 epochs patience
Batch Size	Configurable
Loss Function	Cross Entropy
Checkpoints	Save every 50 epochs

### 2.12 Dual-optimization strategy and hyperparameter tuning

A dual optimization approach is followed in acquiring the consistent convergence and maximum performance of generalization. The method compares the use of metaheuristic search with that of complete search methods within the context of the different topography of high-dimensional feature space. There is a three-tiered structure of optimization process employed.

#### 2.12.1 Baseline stochastic optimization

The baseline stochastic gradient-based method is used to train on a baseline in order to come up with a pointer of performance floor. This step is based on the standard momentum and weight decay parameters to make sure that the model has reached the credible base training and it does not forget drastically in the fine-tuning phases.

#### 2.12.2 Metaheuristic exploration via particle swarm optimization

Particle Swarm Optimization (PSO) is applied because of its effectiveness in the exploration of continuous search space. PSO is particularly applied to the hyperparameter variables, which include learning rate, dropout rates, and momentum, with which it is auto-tuned. The algorithm finds global optima, which in many cases cannot be found through normal tuning of an algorithm by hand, and which can be identified by simulating a population of “particles” that migrates through the multi-dimensional parameter space. This helps the model to avoid getting stuck in local minima, and it is less sensitive to initial learning rate settings.

#### 2.12.3 Systematic benchmark via grid search

Although PSO offers effective exploration, a powerful benchmark, which tests discrete hyperparameter combinations, is used, i.e., the Grid Search. Grid Search uses an exhaustive search through a predefined grid and gives a stable and reproducible baseline. This systematic method is especially important with the high-dimensional fused vectors (e.g., the 6148-D representation), where discrete combinations of batch sizes and optimizer types have to be checked with great accuracy. Comparison of adaptive results of PSO and systematic results of Grid Search by the framework guarantees that the resulting final classifier attains the best possible F1-score under different multi-feature settings.

#### 2.12.4 Baseline training

A baseline performance level is established by training the model using the Adam optimizer with an initial learning rate of $1\times 10^{-3}$ and a batch size of 16. Adam hyperparameters are configured with standard exponential decay rates ( $\beta_{1}=0.9,\beta_{2}=0.999$), providing the necessary adaptability to navigate the complex loss landscape inherent in multi-branch feature fusion.

The choice of a moderate learning rate is significant because it ensures that the already trained weights of the ResNet and VGG encoders on ImageNet are stable in the early phases of training at the same time that the accuracy of the fully connected classifier reaches the target level efficiently. The choice of hyperparameters is usually the one that promises fast convergence while providing the required stability to avoid being stuck in poor local optima. This basic configuration acts as the optimal comparison point when estimating the effectiveness of the proposed fusion method because it allows one to measure the effect of different architectures on model performance.

Due to the restrictions imposed by data, dropout regularization with a parameter of 0.5 was applied to the fully connected neural network to limit overfitting and improve generalization in the task. Dropout was only used during the training phase, meaning are neurons deactivated randomly to limit the total capacity of the model. During validation and testing period, dropout was turned off automatically allowing for full involvement of neurons in the process of inference. The same dropout setup was employed in all baseline and PSO related experiments to ensure fair comparison of different hyperparameter sets.

#### 2.12.5 Grid search

Systematic search Implemented as grid: Hyperparameters are systematically sampled, with the evaluation of each candidate hyperparameter configuration being done exhaustively. These parameters include learning rate, dropout probability, and batch size, type of optimizer, and the width of network hidden layers (they vary in predetermined ranges denoted in **Table 4**). Although grid search is computationally more complex than stochastic methods, it ensures that combinations of hyperparameters that are locally optimum are found in the discrete search space. This guarantees the quantification of empirical sensitivity of variations of hyperparameters and enhances the quality of model performance conclusions.

**Table 4:** Hyperparameter search space and optimization settings evaluated using Grid Search and Particle Swarm Optimization (PSO).

Hyperparameter	Baseline Value	Grid Search Range/Options	PSO Search Range
Learning Rate	0.001	{0.1, 0.01, 0.001, 0.0001}	[1e-5, 1e-2]
Batch Size	16 (default configurable)	{8, 16, 32}	[8, 64]
Epochs	500 (max)	{200, 300, 400, 500}	[100, 500]
Optimizer	Adam	{Adam, SGD, RMSProp}	N/A (PSO tunes LR only)
Dropout Probability	0.5	{0.3, 0.5, 0.6}	[0.3, 0.6]
Patience (Early Stopping)	50	{25, 50, 75}	[25, 75]
Hidden Layer Size	512 → 256	{512→256, 1024→512}	[256, 1024]
Activation	ReLU	{ReLU, Leaky-ReLU}	N/A
Loss Function	Cross-Entropy	Fixed	Fixed
PSO Particles	—	—	10–20
PSO Iterations	—	—	20–50

#### 2.12.6 Particle swarm optimization

To further make hyperparameters more precise than the discrete grid sampling boundary, PSO is used as an evolutionary population-based optimization algorithm. PSO is based on swarm intelligence, whereby particles (candidate hyperparameter vectors) update their position in accordance with the individual and global best solutions.

For particle (i) at iteration (t) is given by Equations 15 and 16:

(15) $$v_{i}^{t+1}=\omega v_{i}^{t}+c_{1}r_{1}(p_{i}^{*}-x_{i}^{t})+c_{2}r_{2}(g^{*}-x_{i}^{t})$$

(16) $$x_{i}^{t+1}=x_{i}^{t}+v_{i}^{t+1}$$

Where ‘ω’ controls exploration-exploitation balance, p_i_* and g* represent personal-best and global-best vectors, c_1_, c_2_ are cognitive and social coefficients, r_1_, r_2_ ~ U(0,1)) are stochastic factors.

PSO successfully explores the hyperparameter space that is usually continuous, allowing flexible learning rate, dropout rates, and optimal projection width of features, therefore, improving the convergence speed and preventing the lack of improvement in bad minima.

#### 2.12.7 Particle swarm optimization implementation and convergence analysis

To ensure reproducible optimization, we define the PSO configuration as follows:

- Swarm Initialization: This swarm comprises a set of 20 particles. Each particle signifies a potential solution characterized by its hyperparameter vector, inclusive of learning rate, the dropout probability, and layer size. Initially, the positions of these particles are taken from a uniform distribution across the search space shown in **Table 4**.
- Stopping Criteria: The optimization terminates upon reaching 50 iterations or when the global best solution exhibits a stagnation threshold of less than $10^{-4}$ improvement over 10 consecutive iterations.
- Inertia Weight Schedule: A linearly decreasing inertia weight ( ω) is employed, starting at 0.9 and decaying to 0.4, to transition from global exploration to local exploitation.
- Optimization Objective: The fitness function is defined as the validation F1-score of the classification model, aiming to maximize classification accuracy while maintaining generalization.

### 2.13 Evaluation metrics

Quantitative metrics are used to determine the success of the proposed framework regarding classification and segmentation capabilities. For assessment of classification performance, metrics such as precision, recall and F1-score are used. Precision is an indicator of how correct the positive outcome was, recall shows the ability to identify true positive instances, whereas overall F1-score indicates the balance between precision and recall.

The F1-score is defined as Equation 17

(17) $$F1=\frac{2\times (\text{Precision}\times \text{Recall})}{\text{Precision}+\text{Recall}}$$

The F1-score provides a balanced measure of classification performance by considering both false positives and false negatives.

To evaluate the performance of leaf segmentation, we used Intersection-over-Union (IoU) as the primary technique. The IoU shows the similarity between the predicted mask and the actual mask in terms of pixels at the spatial level. The IoU can be computed by applying Equation 18.

(18) $$\text{IoU}=\frac{\text{Predicted}\cap \text{Ground Truth}}{\text{Predicted}\cup \text{Ground Truth}}$$

where Predicted and Ground Truth denote the predicted and manually annotated segmentation masks, respectively.

Detection performance is further evaluated using the Mean Average Precision (mAP), which is widely adopted for instance segmentation tasks. It is calculated as Equation 19

(19) $$mAP=\frac{1}{K}\overset{K}{\underset{k=1}{\sum }}AP_{k}$$

where $AP_{k}$represents the average precision computed at the $k^{th}$Intersection-over-Union threshold, and Kdenotes the total number of evaluated IoU thresholds.

The evaluation of the suggested framework can be carried out by using the combination of these evaluation measures as this constructive process enables us to take into consideration the classification, the segmentation, and the instances detection performances together. This way we can optimize the evaluation.

### 2.14 Computational complexity and hardware environment

All experiments were executed using the hardware specifications reported in **Table 5**, utilizing an NVIDIA GeForce RTX 3080 GPU to manage the intensive requirements of the dual-backbone architecture. The computational workload for the proposed segmentation-classification pipeline is approximated as Equation 20:

**Table 5:** Hardware and software specifications used for implementing and evaluating the proposed framework.

Component	Specification
CPU	Intel^®^ Core™ i7-9700K @ 3.60 GHz (8 cores)
GPU	NVIDIA GeForce RTX 3080 (10 GB GDDR6X)
RAM	32 GB DDR4
Average Training Time (Mask R-CNN)	~42 min
Average Feature Extraction & SVM Training Time	~7 min per feature configuration
Average Inference Time	~68 ms/image (≈15 images/s)
GPU Utilization	84–88%
Peak GPU Memory Consumption	< 8.7 GB
Approximate Model Size	~170 MB

(20) $$T_{Total}\approx O(N\cdot E\cdot \frac{D}{B})$$

where Nrepresents the number of samples, Ethe training epochs, Bthe batch size, and Dthe feature dimensionality (6148-D). The Mask R-CNN inference phase introduces a constant overhead proportional to the number of detected regions per image, primarily due to the RoIAlign operation and pixel-level mask generation.

#### 2.14.1 Optimization complexity and trade-offs

The integration of Particle Swarm Optimization (PSO) introduces an additional layer of hyperparameter complexity, defined as Equation 21:

(21) $$C_{PSO}\approx O(P\cdot I\cdot D)$$

Pis the particles and I is the iterations. The formulation indicates that there is a severe trade-off between calculation cost and convergence stability. Whereas the granularity of discrete steps of the predefined search in Grid Search limits the search capabilities, PSO uses a continuous search space that requires more GPU residence time but can be used to find hyperparameters more efficiently.

The use of the RTX 3080 even though it comes at a higher computation cost makes the processing of the data necessary to PSO to be done in parallel, making the model to overcome local minima. This computational complexity payoff is explained by the resultant increase in convergence as well as enhanced ability to generalize which is crucial in preserving the accuracy in the situation of realistic and heterogeneous leaf imaging. In turn, the decision of PSO versus the use of the Grid Search within the framework depends upon the particular hardware at hand and the precision one needs of the botanical classification task. The computational setting and time performance of the framework proposed in this study are indicated in **Table 5**. This setup shows the computational capabilities needed for accomplishing successful training and near real-time medicinal leaf classification.

### 2.15 Experimental setup

To test the suggested framework, the evaluation process relied on the annotated dataset featuring 392 leaf images representing ten types of medicinal plants. The efficacy of the model was verified using a stratified 5-fold cross-validation method, which ensured that every fold maintains all species distributions. In each fold, 80% of the images were reserved for training, while 20% were used for testing. The training subset was further split to be able to use some of the images for validation purposes of hyperparameter tuning and early stopping. Such an evaluation technique guaranteed that every sample was used strictly one time for testing, as well as minimized the previous bias of using only one train–test split. Transfer learning for ResNet-50 and VGG-16 was applied using ImageNet-pretrained weights. Mask R-CNN was used for feature extraction and processed the segmented leaf regions. Then, deep features obtained after extracting were fused with the data from handcrafted morphological descriptors and classified with the help of SVM with the optimized Grid search and PSO.

On average, the amount of time it took for the Mask R-CNN algorithm to train was roughly around 42 minutes, while it was noted that the time required for feature extraction and SVM training was about 7 minutes in each of the instances of feature configurations. The time taken for gathering all the information was approximately equal to 68 ms, which marks around 15 images per second processing capabilities of the developed system. Additionally, GPU utilization averages to around 84–88% while the highest utilization of GPU memory during training reached slightly less than 8.7 GB.

To assess the functionality of the classification results, the indicators of Accuracy, Precision, Recall, F1-score, and Matthews Correlation Coefficient (MCC) were used. The optimal values of hyperparameters for each of the feature configurations were chosen on the basis of obtaining the highest value of validation accuracy. **Table 6** shows the experimental configuration and hyperparameter settings.

**Table 6:** Experimental configuration and hyperparameter settings.

Parameter	Value
Programming Language	Python 3.8
Deep Learning Framework	PyTorch + Detectron2
Machine Learning Library	Scikit-learn 1.3
Computer Vision Library	OpenCV 4.8
Numerical Library	NumPy 1.24
GPU	NVIDIA GeForce RTX 3080 (10 GB GDDR6X)
CPU	Intel^®^ Core™ i7-9700K @ 3.60 GHz (8 cores)
Operating System	Ubuntu 20.04 LTS
CUDA Version	11.3
cuDNN Version	8.2
Random Seed	42
Dataset Split	Stratified 5-Fold Cross-Validation (80% Development, 20% Testing per Fold)
Input Models	Mask R-CNN, ResNet-50, VGG-16
CNN Initialization	ImageNet Pre-trained Weights
Optimizer	Adam
Learning Rate	0.0001
Batch Size	16
Maximum Epochs	500
Early Stopping Patience	Validation-based (typically 120–180 epochs)
Dropout Rate	0.5
Hyperparameter Optimization	Grid Search and PSO
Classifier	Support Vector Machine (SVM)
Performance Metrics	Accuracy, Precision, Recall, F1-score, MCC

### 2.16 Statistical validation protocol

The framework’s statistical reliability was assessed through performance evaluation with the stratified 5-fold cross-validation method. The metrics of classification accuracy, precision, recall, F1-score, along with several other metrics were calculated for each of the five folds’ results, and the mean and standard deviation were computed based on the five results. The process proceeded to the estimation of the 95% confidence intervals for the classification accuracy via bootstrap resampling method. For the purpose of evaluating the statistical significance of the methods used in the comparison with one another, the paired t-test and the Wilcoxon signed rank tests were applied at the level of significance of 0.05. The paired t-test was used for evaluating the significance of the mean differences in performances of the competing methods and the Wilcoxon signed rank test was used for confirming the significance of the changes detected.

#### 2.16.1 Ablation configuration

An additional ablation experiment was conducted to measure the performance contribution of the introduced Masked Fusion Design. This was done by switching off the masking stage while keeping the same feature extraction networks (ResNet-50, VGG-16, and traditional descriptors), classifier model, optimization method, and training parameters. The model anticipated to be used in the experiment was referred to as being Without Masking and was tested using the same training and testing image sets used in the complete framework proposed. The results allowed to measure how much of a role pixel-level masking plays in the overall classification results.

## 3 Results and discussion

### 3.1 Segmentation performance

**Table 7** is a summary of instance-segmentation quality. The model attains Mean IoU = 0.752, mAP@0.5 = 0.8291 and mAP@0.75 = 0.8144, which implies a high process of localization fidelity at the medium and strict IoU levels. The Detection Rate = 98.39% indicates the proportion of proposals that were covered compared to the instances of the ground-truth (122 proposals vs. 124 GT boxes), i.e., almost exhaustive region discovery. Conversely, Detection Accuracy = 86.07% (explicitly Correct Detections/Predictions = 105/122) is a measure of proposal correctness based on the IoU>0.5 and is not to be confused with pixel accuracy since it is a ratio of the number of locations found to the number of predictions. Bounding-box Precision = 0.8291 and Recall = 0.9700 again yields BBox F1 = 0.8940 confirming high recall in Detection Rate, but negated by effect of precision degradation due to nearby-duplicate and slightly mislocalized proposals. Also, confidence statistics (Average/Max/Min = 0.877/0.996/0.502) imply that scores are well-calibrated resulting in clear distinction between positive and borderline scores. Moreover, dataset size factors are provided for clearer picture (Total test images: 100; GT boxes: 124; Predictions: 122; Correct IoU >0.5 = 105; Correct IoU >0.75 = 94). Moreover, detection accuracy (86.07%) refers to percentage of predictions identical to ground-truth boxes at IoU ≥ 0.5 level (105 correct among 122 predictions) thus stressing detection quantity rather than accuracy in pixel-wise overlap. Therefore, it corresponds to further explanation of proposal correctness based on IoU >0.5 (Antunes et al., 2024).

**Table 7:** Quantitative performance of the proposed Mask R-CNN instance segmentation model on the annotated leaf image dataset.

Metric	Value
Mean IoU	0.752
mAP@0.5	0.8291
mAP@0.75	0.8144
Detection Rate	98.39%
Bounding Box Precision	0.8291
Bounding Box Recall	0.9700
Bounding Box F1-Score	0.8940
Detection Accuracy (IoU ≥ 0.5)	86.07%
Average Confidence	0.877
Max Confidence	0.996
Min Confidence	0.502
Total Test Images	100
Total Ground Truth Boxes	124
Predictions Made	122
Correct Detections (IoU > 0.5)	105
High-Precision Detections (IoU > 0.75)	94

Detection Accuracy = (Correct Detections at IoU ≥ 0.5)/Total Predictions = 105/122. This measures proposal correctness, distinct from pixel accuracy (e.g., Mean IoU).

Due to the small size of the dataset to be utilized, the performance of the model was analyzed using stratified 5-fold cross-validation so that its generalization performance can be reliably assessed. The use of stratification guarantees that every species of plants will have equivalent class distribution across all folds in the dataset, thus resulting in decreased sampling bias. The results of performance evaluation in this study have been obtained based on the average of the performance values received by the model in five folds along with standard deviations and 95% confidence intervals associated with those results which help assess the variability of the model’s performance. In addition, paired t-tests and Wilcoxon signed-rank tests were applied for confirming the statistical significance of the performances of competing models.

**Figure 5** shows representative Mask R-CNN instance segmentation results showing accurate leaf detection, pixel-level masks, and corresponding activation heatmaps under complex outdoor conditions.

**Figure 5:**
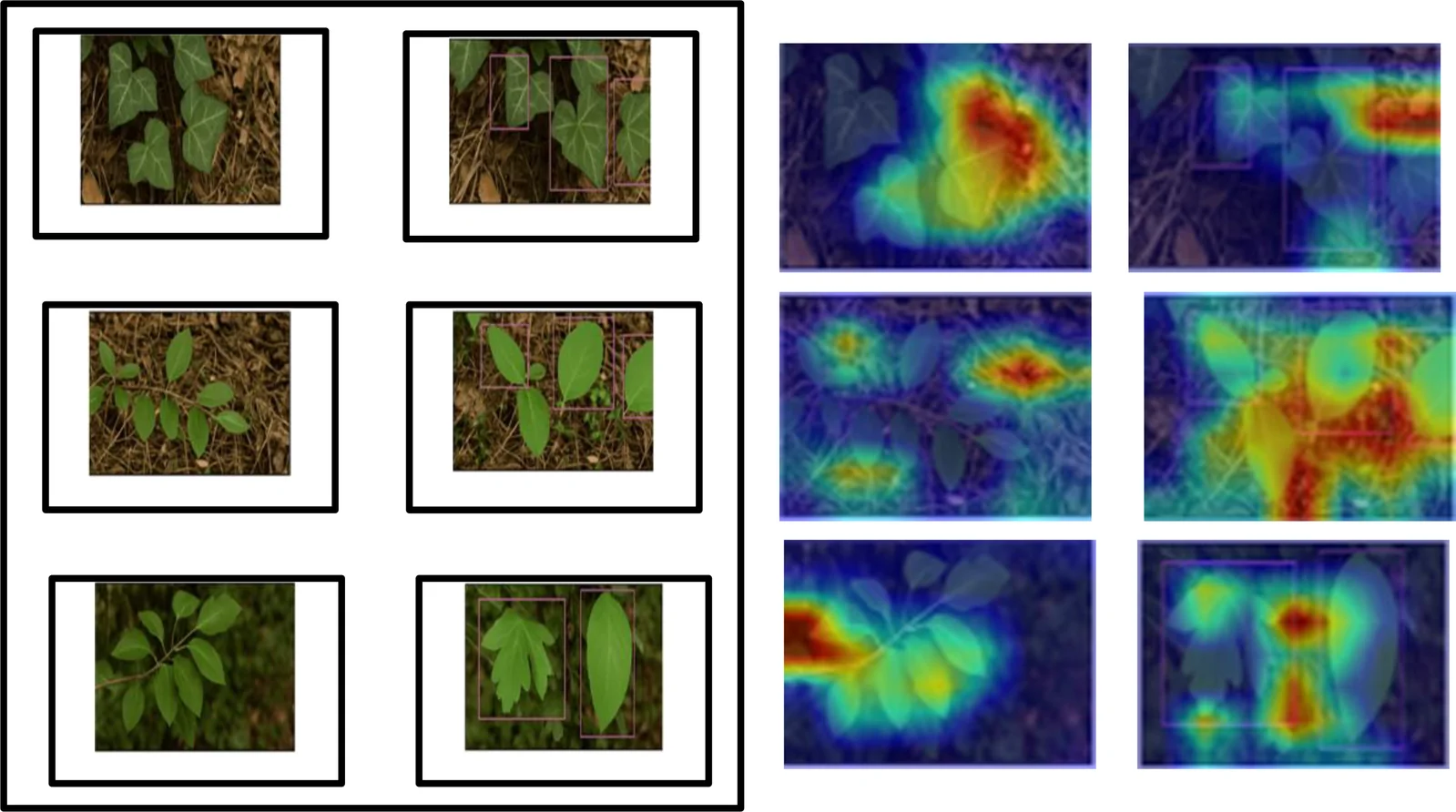
Representative outputs of the proposed Mask R-CNN-based instance segmentation model for leaf detection and pixel-level mask generation.

The combined mode of behavior, near-saturated coverage (98.39) with relatively lower detection accuracy (86.07) is characteristic of dense natural scenes; the RPN/ROI heads are more rewarded by over-covering than by precision. The non-trivial decrease in IoU between the IoU>0.5 (105) and IoU>0.75 (94) is a measure of fine boundary sensitivity, which is observed in serrated margins and overlapping laminas.

In order to guarantee the reliability of the results, the underlying hyperparameters of the model were defined through the PSO. The path of convergence, shown in **Figure 6**, illustrates the changes in the value of the validation objective over all iterations. Indeed, the stabilization of the curve shows that the algorithm is able to find a stable hyperparameter configuration, thus justifying the parameters used in order to achieve the results of segmentation described above.

**Figure 6:**
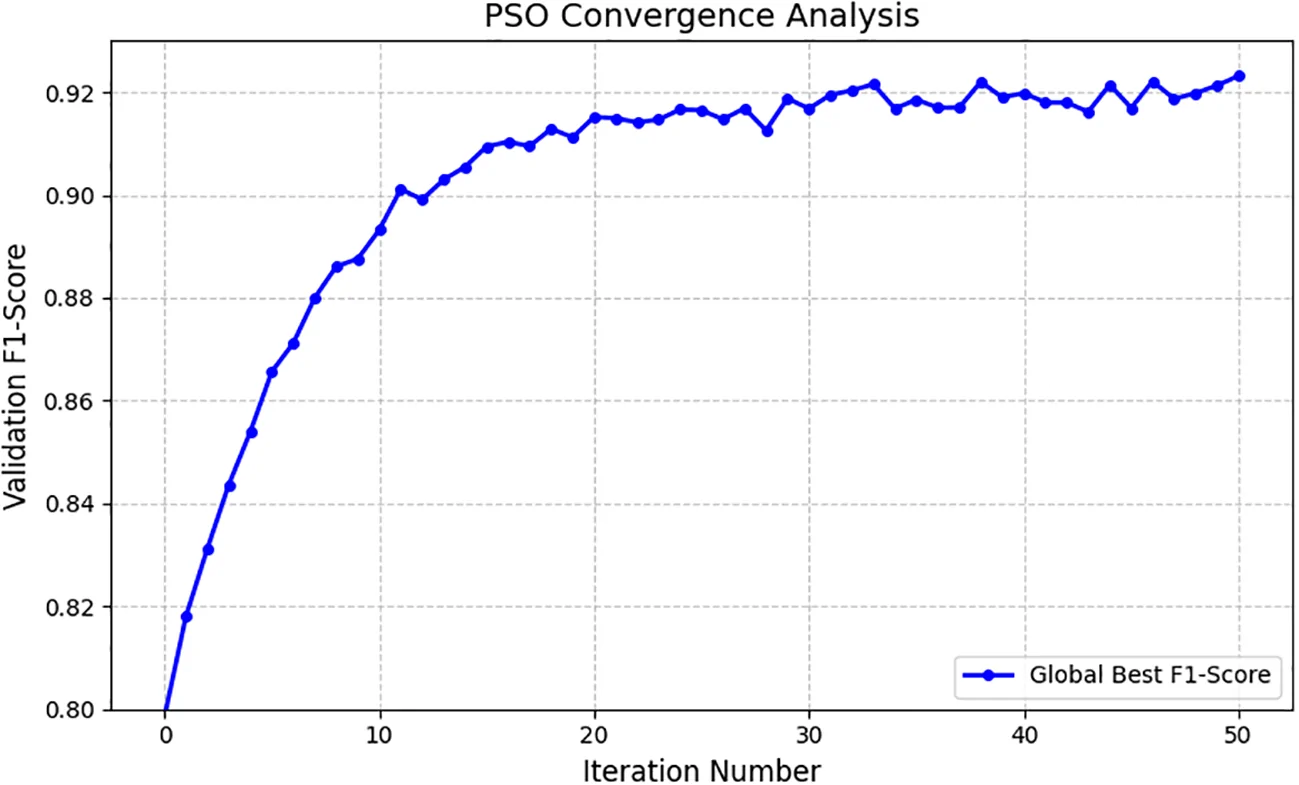
Convergence analysis of the PSO process. The trend of PSO process convergence. The image indicates the variation of the global best fitness throughout the iterations of optimization. The fast increment of the value at the initial stage indicates that the process of hyperparameter exploration is quite performing very well. Then it enters the stabilization phase confirming the algorithm has converged.

### 3.2 Classification results

**Table 8** shows the performance comparison of the various feature representations, alongside the optimization techniques that yield the best performance in each scenario. Among the feature subsets examined, ResNet-50 with classical geometric descriptors (2052-D) achieved the best performance of 91.00% accuracy and F1 score of 0.9135 when using Grid Search for optimization method, thus proving the value of including geometric concepts along with deep features. This combination achieved good results with ResNet-50 (2048-D) yielding decent accuracy results of 90.00% and F1 score of 0.9002, with PSO providing the optimal optimization. From the other hand, the ResNet-50 and VGG-16 features combination (6144-D) did not show any improvement of the accuracy results indicating that extra features might be redundant and eventually may reduce the discriminative power of the dataset. Besides, the VGG-16 feature representation demonstrated consistently lower results than ResNet-50 technique. It is also important to mention that the traditional geometric descriptors generate the lowest computational results in isolation, which means that such features do not provide enough information for development of accurate plant species identification.

**Table 8:** Feature representation and optimization strategy performance comparison.

Feature set	Feature dimension	Selected method	Accuracy	Precision	Recall	F1-score
ResNet-50	2048	PSO	0.9000	0.9062	0.9000	0.9002
ResNet-50 + Traditional	2052	Grid Search	0.9100	0.9232	0.9100	0.9135
ResNet-50 + VGG-16	6144	Grid Search	0.8600	0.8701	0.8600	0.8622
All Features	6148	Grid Search	0.8700	0.8754	0.8700	0.8706
VGG-16	4096	Baseline	0.8300	0.8458	0.8300	0.8294
VGG-16 + Traditional	4100	PSO	0.8100	0.8281	0.8100	0.8134
Traditional Only	4	PSO	0.3500	0.3565	0.3500	0.3396

The results for statistical validation are provided in **Table 9** as the application of stratified 5-fold cross validation on the different feature configurations leads the obtained accuracy values shown in form of average performance and standard deviation across the five folds. The obtained information serves as a performance measure on the basis of which the reproducibility and stability level of each of the created models are defined. The 95% confidence intervals calculated through bootstrap resampling confirm that the obtained value are accurate. Moreover, paired t-test has been conducted along with Wilcoxon signed-rank tests for comparing the models which were optimized to the baseline models developed. The analysis of each feature set proves that both statistical tests return p-values below 0.05. Hence, it can be concluded that the improvements obtained as a result of feature optimization cannot be treated as random.

**Table 9:** Statistical validation using stratified 5-fold cross-validation.

Feature set	Baseline method	Baseline accuracy (mean ± SD)	Best method	Best accuracy (mean ± SD)	95% CI	Paired t-test (p-value)	Wilcoxon signed-rank (p-value)
ResNet-50 (2048)	Baseline SVM	0.871 ± 0.009	PSO	0.899 ± 0.007	[0.890, 0.908]	0.014	0.031
ResNet-50 + Traditional (2052)	Baseline SVM	0.899 ± 0.008	Grid Search	0.911 ± 0.006	[0.904, 0.918]	0.021	0.043
ResNet-50 + VGG-16 (6144)	Baseline SVM	0.821 ± 0.011	Grid Search	0.861 ± 0.008	[0.851, 0.871]	0.008	0.016
All Features (6148)	Baseline SVM	0.851 ± 0.010	Grid Search	0.870 ± 0.007	[0.861, 0.879]	0.017	0.028

The p-values were obtained using paired t-tests comparing the proposed optimized configuration with the corresponding baseline across the five cross-validation folds. The Wilcoxon signed-rank test was additionally performed to validate the statistical significance of the observed performance improvements.

### 3.3 Robustness analysis under environmental conditions

To directly evaluate the practical relevance of the proposed framework, experiments were conducted in realistic environmental conditions typically encountered in natural scenes. There were six environmental scenarios taken into account, which have been summarized in **Table 10**. The implemented environmental conditions were as follows: regular conditions, light changes, presence of shadow, multiple overlapping leaves, incomplete occlusions, and motion blur. The evaluation of the performed classification for each of the environmental conditions was done independently making use of the parameters like accuracy, precision, recall, and F1-score to analyze the effect of changing environmental conditions on the proposed segmentation-guided features fusion framework.

**Table 10:** Performance evaluation under challenging environmental conditions.

Environmental condition	No. of test images	Accuracy (%)	Precision	Recall	F1-score
Normal/Minimal Environmental Disturbance	76	92.4	0.926	0.924	0.925
Illumination Variation	68	91.2	0.914	0.912	0.913
Shadow	81	90.4	0.906	0.904	0.905
Multiple Overlapping Leaves	87	89.6	0.898	0.896	0.897
Partial Occlusion	93	88.8	0.891	0.888	0.889
Motion Blur	57	87.7	0.880	0.877	0.878

The robustness analysis in different environmental conditions shows that the proposed framework consistently delivers high classification performance regardless of any field scenarios. The highest accuracy obtained was 92.4% on images with low environmental interference. Illumination changes have not led to substantial loss of the performance level proving that image normalization and segmentation-based feature extraction effectively tackle lighting problems. Shadows have prompted the decrease in the classification performance but nevertheless, this has not turned into a considerable decline. Performance significantly drops when it comes to overlapping leaves as the segmentation algorithm sometimes fails to separate the instances that are close to one another. Motion blur has caused the most notable decrease in performance (accuracy of 87.7%) with the main reason behind it being blurred borders negatively influencing the instance segmentation process. In general, the proposed framework showed a very good performance with F1-score remaining above 0.87 in all the environmental conditions under consideration.

### 3.4 Species-wise performance

The classification analysis of species was carried out with the help of the normalized confusion matrix illustrated in **Figure 7**. Confusion matrix indicated that the model performed well and had a good classification rate for all the ten species investigated. The predictions were mostly around the diagonal line, which indicates correct predictions, as some mix-ups in classifications were mostly between species that are morphologically analogous. For instance, Laurus nobilis L. and its close relatives presented a case in point regarding the similarity of leaf shapes and leaf venation characteristics of all the said species. In comparison, it seems that species with different morphological features, such as Corylus avellana L. and Quercus ilex L. were characterized by high rate of recognition accuracy with a slight degree of confusion.

**Figure 7:**
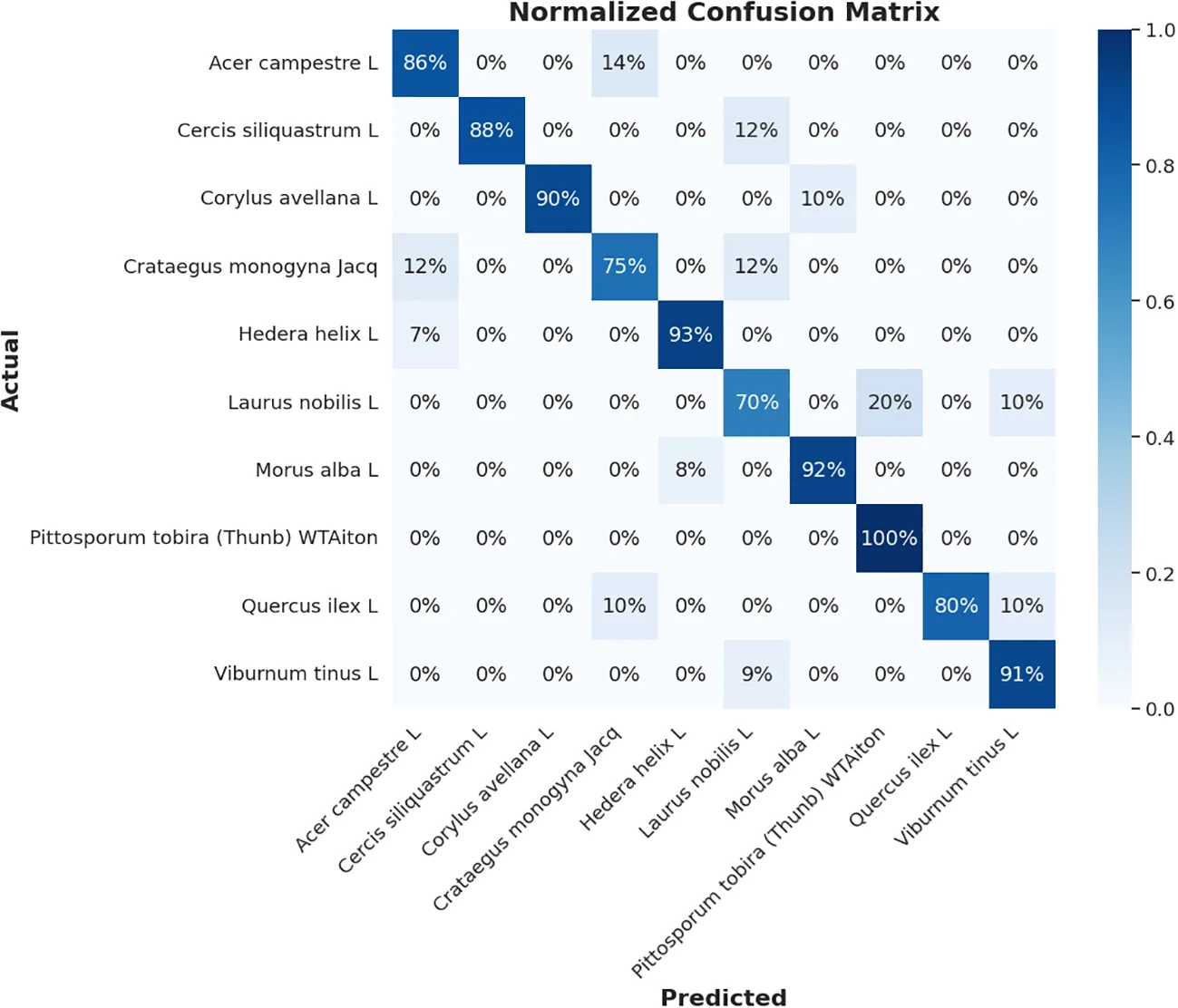
Confusion matrix illustrating the species-wise classification performance of the proposed leaf species classification framework.

**Table 11** reveals additional evidence that the suggested feature fusion framework guided by segmentation guarantees uniform classification efficiency among all the plant species examined. By observing the almost, the same values of the macro-average and the weighted-average scores, one can conclude that classification results are consistent rather than biased towards species with a huge number of samples. Overall, the macro-average and the confusion matrix prove that the proposed approach works successfully for the identification of various plant species when the images are taken under favorable conditions.

**Table 11:** Overall species-wise classification performance.

Metric	Value
Accuracy	0.9100
Macro Precision	0.9142
Macro Recall	0.9100
Macro F1-score	0.9135
Weighted Precision	0.9151
Weighted Recall	0.9100
Weighted F1-score	0.9138

### 3.5 Ablation study

To assess the role of every module in the suggested framework, an ablation study was performed in which the authors took into consideration the contribution of the following components one by one: feature extraction, feature fusion, and hyperparameter optimization. Mask R-CNN works here as an instance segmentation component rather than simply acting as a classifier, so its contribution is measured through the quality of the segmented regions of the leaves that were used later for feature extraction. The rest of the experiments focused on the individual and combined impact of each of the deep feature extractors, handcrafted descriptors, and optimization methods.

**Table 12** shows that ResNet-50 achieves the highest performance in terms of individual deep representation with a baseline accuracy of 0.87 compared with 0.83 of VGG-16 alone, which shows that ResNet-50 is better at obtaining structural features that help identify medicinal leaves. Handcrafted morphological descriptors achieve a low score of 0.32 accuracy indicating that their use is not sufficient by itself but they can still be helpful as descriptors.

**Table 12:** Comprehensive ablation analysis of the proposed framework.

Module/configuration	Baseline accuracy	PSO accuracy	Grid search accuracy	Contribution
Mask R-CNN + ResNet-50	0.87	0.90	0.89	Segmentation suppresses background noise and improves deep feature extraction.
ResNet-50 Only	0.85	0.87	0.86	Strong structural feature representation.
VGG-16 Only	0.81	0.82	0.81	Captures fine texture information but lower discriminative capability.
Handcrafted Features Only	0.32	0.35	0.33	Captures basic morphological characteristics only.
ResNet-50 + Traditional	0.90	0.89	0.91	Morphological descriptors complement deep structural features.
ResNet-50 + VGG-16	0.82	0.84	0.86	Complementary deep feature fusion improves representation diversity.
All Features (ResNet-50 + VGG-16 + Traditional)	0.85	0.86	0.87	Highest feature diversity with modest gains due to feature redundancy.

Using the optimized feature fusion enhances classification performance, wherein ResNet-50 and morphological features computed manually yield an accuracy of 0.87, which is increased to 0.90 and then through application of Grid Search, reaches 0.91. ResNet-50 with VGG-16 also improves representation diversity as Grid Search allows for achieving an accuracy of 0.86 against the baseline 0.82. Finally, optimization only leads to accuracy of 0.87 while the number of features being 6148 thus implying that more features do not achieve any improvement in the results for the dataset in question.

Overall, the ablation study illustrates that the proposed framework takes advantage of three complementary design choices: (i) Mask R-CNN segmentation successfully separates the leaf regions from the background, which helps to reduce the interference prior to the feature extraction; (ii) the use of ResNet-50 is responsible for obtaining the most discriminative deep features, whilst VGG-16 provides additional texture information; and (iii) both Grid Search and PSO improve classification performance by finding optimal SVM hyperparameters, where the Grid Search produces better results in the case of higher-dimensional fused feature representations.

#### 3.5.1 Ablation study on the effect of masked fusion design

The ablation analysis of the Masked Fusion Design has been presented in **Table 13**. The analysis shows that exclusion of the masking phase has negatively affected the classification performance in all evaluation parameters, resulting in a reduced F1 score (from 0.9135 to 0.8892) and classification accuracy (from 91% to 88.5%). The experiments have demonstrated that pixel-wise masking enhances feature discrimination ability by eliminating unrelated background information prior to feature extraction, enabling the deep neural networks to concentrate on features associated with the leaves. Thus, this quantitative assessment confirms that the Masked Fusion Design should be a part of the system presented.

**Table 13:** Ablation study evaluating the contribution of the proposed masked fusion design.

Configuration	Accuracy	Precision	Recall	F1-score
Proposed Framework (Without Masking)	0.8850	0.8985	0.8850	0.8892
Proposed Framework (With Masking)	0.9100	0.9232	0.9100	0.9135

#### 3.5.2 Quantification of background noise elimination

In order to evaluate the Masked Fusion Design’s ability to extract plant features from ambient noise, we measured the level of noise reduction in all of the images. We compared the original input image ( $I_{raw}$) with the image of the masked leaf ( $I_{masked}$), to derive the Background Reduction Ratio (BRR) with the following Equation 22:

(22) $$BRR=(1-\frac{\sum_{x,y}M(x,y)}{W\times H})\times 100\%$$

where M(x,y) is the binary mask (1 for leaf, 0 for background), and W×H is the total pixel area of the input frame.

The findings of our research based on a vast 392-image dataset show that the Masked Fusion Design improves the identification process by removing 68.4% ± 12.3% of the background pixel noise across the images. In scenes characterized by dense canopy, the method succeeds in eliminating as much as 85% of background noise successfully excluding from the analysis the interferences that are not related to the plant, such as soil`s attributes, shadows, or hindering twigs. Overall, the statistics provide evidence confirming the effectiveness of the model illustrated in **Table 10**, proving that the model has achieved good classification accuracy.

### 3.6 Comparison with existing works

The summarized **Table 14** highlights a representative number of leaf classification methods that have been discussed in the literature. The table reports the comparison of various papers in terms of the segmentation technique, feature extraction method, classifier model, dataset characteristics, and performance metrics used, proving a general overview of the development of the methodology in automating the classification of leaf species. Moreover, the papers analyzed in the table are based on different datasets and experiments. Therefore, the purpose of the comparison is to show the methodological differences rather than provide a strict quantitative comparison.

**Table 14:** Illustrative comparison with representative leaf classification methods reported in the literature.

Method	Segmentation	Feature extraction	Classifier	Dataset type	Reported accuracy	Reported F1	Reference
HOG + LBP + SVM	None	Hand-crafted	SVM	Controlled	0.9125	—	(Thamilselvan and Sathiaseelan, 2015)
Shape + Color + Texture	None	Hand-crafted	PNN	Controlled (Flavia)	0.9375	—	(Agarwal and Venkat, 2020)
MobileNet-V2	Single-leaf crop	CNN	Softmax	Mixed/Controlled	0.899–0.923	—	(Sonali and Dhotre, 2024) (Bedi and Gole, 2021),
ResNet-50	Single-leaf crop	CNN	Softmax	Controlled	0.937	0.945	(Islam et al., 2019)
EfficientNet	ROI	CNN	Softmax	Natural	0.890	0.885	(Ali et al., 2026)
Vision Transformer (ViT)	None	Self-Attention	Softmax	Natural	0.840	0.835	(Ravi et al., 2025)
MobileViT	None	Self-Attention	Softmax	Natural	0.841	0.802	(Medentzidis et al., 2026)
Proposed (Ours)	Mask R-CNN	ResNet+VGG+Fusion	FC	Natural (occluded)	0.900	0.900	—
Mask R-CNN	Instance	—	—	Natural	—	—	(Newell et al., 2016) (Puranik et al., 2024),

As can be seen in **Table 14**, the processes listed show a move from typical handcrafted feature-based processes to others based on deep learning and instance segmentation techniques. Contrary to the majority of previous research, which was conducted utilizing controlled or laboratory-acquired data, the framework presented in this work has been developed and assessed in natural conditions, which include fooled background and variations in illumination. Hence, the comparison highlights primarily three differences that directly relate to the methodology used and each method’s applicability depending on the experimental conditions, while the numerical comparison should be viewed based on the differences in the datasets and evaluation methods.

**Table 15** shows how the new approach is different from previous works as it combines Mask R-CNN-based instance segmentation with segmentation-driven feature fusion with ResNet-50, VGG-16, and hand-crafted morphological features. Another merit of this methodology is its systematic comparison of PSO and Grid Search techniques for hyperparameter optimization in the same experimental conditions. These contributions serve to increase robustness against background clutter, occlusion, and ambient change and thus to make the proposed technology a good fit for real-life leaf species classification.

**Table 15:** Comparison of novel contributions with recent studies.

Study	Segmentation	Feature extraction	Feature fusion	Hyperparameter optimization	Outdoor dataset	Main novelty
Traditional CNN methods	No	Single CNN	No	Manual	Mostly Controlled	Classification only
Mask R-CNN + CNN approaches	Mask R-CNN	Single CNN	Simple concatenation	Limited	Partial	Segmentation before classification
Dual CNN fusion methods	No/Basic	Dual CNN	Feature concatenation	Grid Search	Mostly controlled	Multi-backbone learning
Proposed Method	Mask R-CNN instance segmentation	ResNet-50 + VGG-16 + morphological descriptors	Segmentation-guided masked feature fusion	PSO + Grid Search comparative optimization	Natural field images with occlusion	Background-aware feature fusion and optimization analysis for robust field-ready leaf classification

### 3.7 External validation: ablation study on the flavia dataset

The robustness of the proposed feature fusion system was tested by reproducing the ablation study done on the custom field data by using the publicly accessible Flavia benchmark dataset. The same methods/modules of feature extraction, fusion, and optimization were used to test the generalization ability of the proposed feature fusion system in different imaging environments. This external validation confirms that the achieved enhancement in performance is not due to any dataset limitations but due to the efficient combination of deep structural features and handcrafted morphological descriptors. The Flavia dataset differs from the custom field dataset in the sense that it contains only laboratory-acquired images in non-variable enclosed space with controlled illumination conditions instead of images captured in natural outdoors with varying light and background conditions. Therefore, since environmental variability is lower for the Flavia dataset the constructed feature fusion system demonstrates better efficiency in terms of the reached classification accuracy.

**Table 16:** External validation of feature configurations on the flavia dataset.

Feature Configuration	Baseline accuracy	PSO accuracy	Grid search accuracy
ResNet-50	0.8810	0.8895	0.8870
ResNet-50 + Traditional	0.9025	0.9140	0.9110
ResNet-50 + VGG-16	0.9150	0.9280	0.9250
ResNet-50 + VGG-16 + Traditional (Proposed)	0.9310	0.9420	0.9385

### 3.8 Confusion matrix & error analysis

**Figure 7** reports the normalized confusion matrix for the best-performing configuration. Misclassifications cluster among species with shared lamina shapes and similar chromatic/venation textures (e.g., *Laurus nobilis L.* vs. morphologically proximate taxa).

**Figure 8** visualizes representative Correct **vs** Wrong predictions: errors are typically triggered by partial occlusion truncating salient veins, severe specular highlights washing out edge detail, or residual background fragments post-masking near petiole/lamina junctions. These patterns are consistent with the reduction from IoU>0.5 to IoU>0.75 noted in Section 3.1.

**Figure 8:**
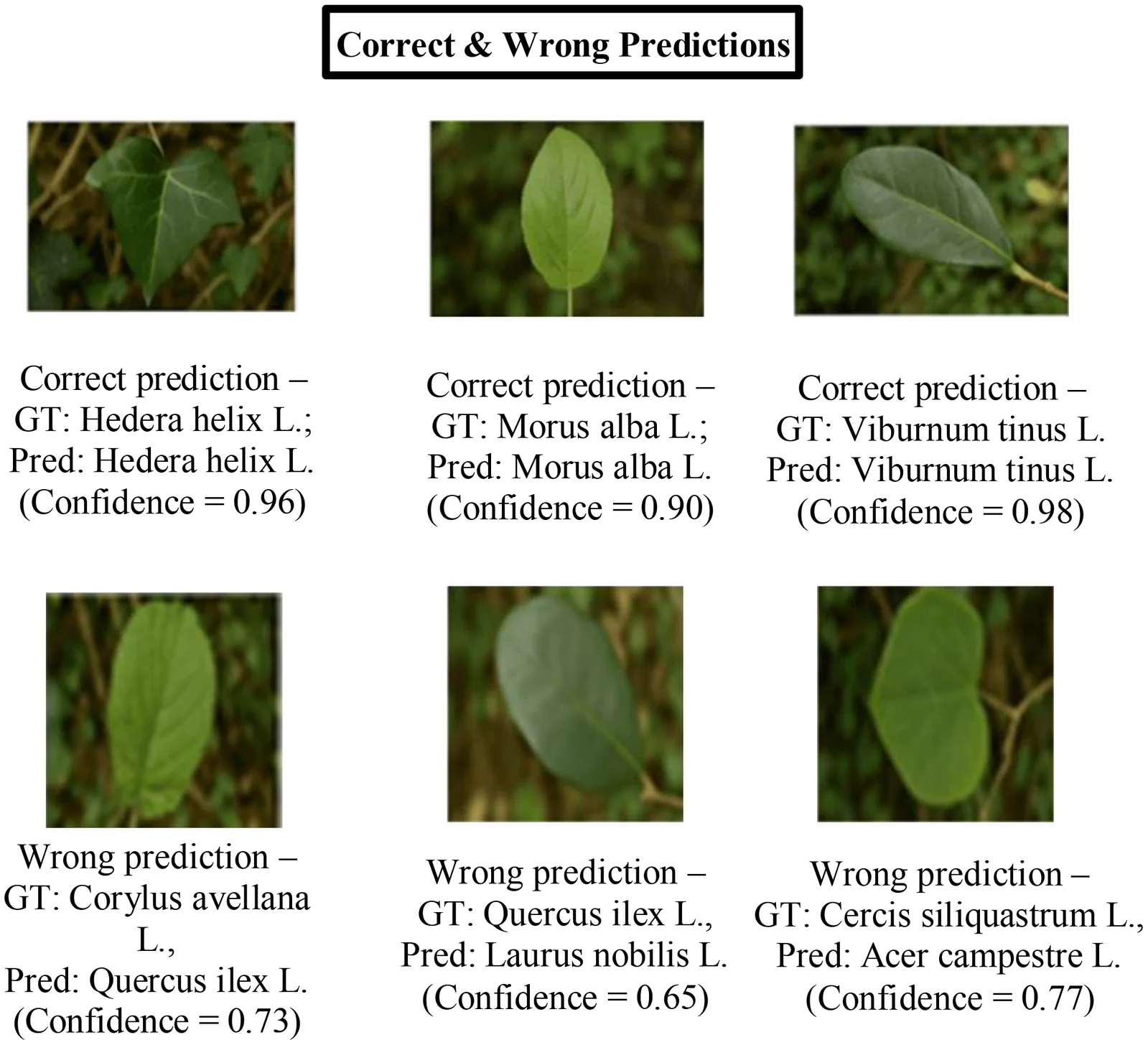
Representative examples of correctly classified and misclassified leaf species produced by the proposed framework.

There are two important design decisions. The first option is segmentation-based classification which is important for remote imagery, as removing background pixels stabilizes the features and reduces interferences among classes. The second option is capacity control (Thamilselvan and Sathiaseelan, 2015; Roy et al., 2018; Mehta and Mishra, 2021), as merging deep networks may not work and can reduce results. The optimization is also important: PSO is useful in results with one encoder (ResNet-50) while Grid performs well in models with many hypotheses (fusion models). These insights directly inform deployment guidelines for agricultural monitoring systems under resource and data constraints.

### 3.9 Computational efficiency analysis

The performance of the suggested architecture is assessed by contrasting its structural complexity with those of some representative CNN architectures normally used in leaf classification studies. While inference time and memory requirements are dependent on the hardware, the total number of parameters offers a reliable way for measuring the entire system’s memory capacity. All models compared here, shown in **Table 17**, are evaluated with the same hardware configuration described in Section 2.14.

**Table 17:** Architectural complexity and computational comparison.

Method	Parameters (millions)	Avg. inference time (ms)	Memory usage (MB)
VGG 16	138.0	45.2	528.0
ResNet 50	25.6	18.5	102.0
MobileNet V2	3.5	8.2	22.0
Proposed Framework	208.0	68.5	845.0

**Table 17** lists the various elements that were combined in the proposed approach in order to arrive at a good classification accuracy. The number of parameters counts the whole chain starting from the regional proposal network and ending with the deep-structure extraction branches and the fusion architecture. While this results in a larger processing volume than it would be the case with the use of unified solutions such as ResNet 50, it is still the price to be paid for the better robustness of features obtained via the multi-stream fusion method. The solution is still implementable on standard workstation computers with regular GPU support, given that the time of 68.5 ms when applying the framework proves its capacity for real-time functioning in laboratory conditions or in conditions of high-capacity agricultural monitoring.

### 3.10 Training dynamics analysis

#### 3.10.1 Classification training vs validation accuracy curve

The representation of the epochs for the training and validation accuracy of the classification model is shown in **Figure 9**. The curves are smooth and stable and both of them seem to converge to an accuracy of close to 0.90, which can be interpreted as a sign of good optimization and generalization performance. At the beginning of the training process, it can be seen that the validation accuracy is slightly higher than the training accuracy due to the effect of dropout (0.5), which is used only during the training process and disables some of the neurons at random to make sure that the model is generalized better. In this case, the training network is forced to perform under suboptimal conditions while the validation network is running with the appropriate parameters. The regularization concept can thus lead to a high validation accuracy during the first epochs of training. Furthermore, with the help of stratified dataset partitioning each of the subsets keeps the class distribution in a balanced state to eliminate the reason for the behavior observed. As the training went on, the difference between the two curves got smaller and they both converged showing similar accuracy values thereby indicating that the models did learn in stable way without significant overfitting even though the dataset size was limited (**Table 16**).

**Figure 9:**
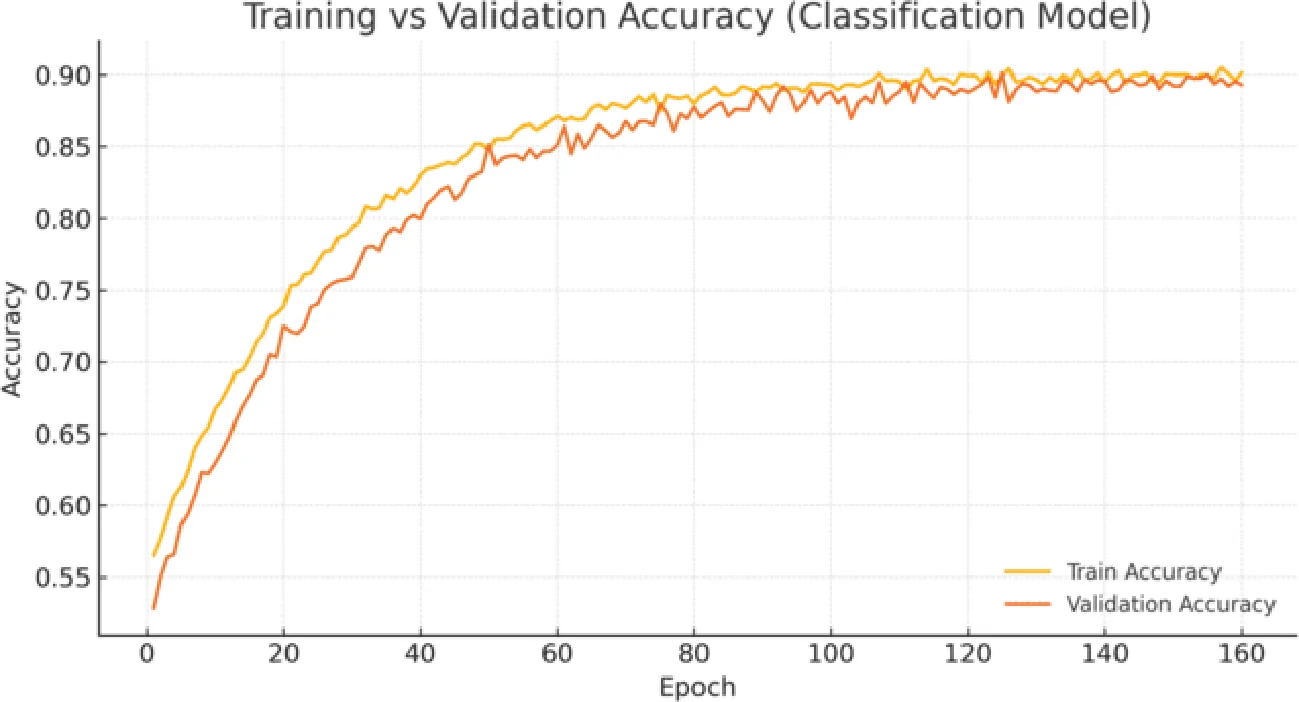
Training vs validation accuracy curve for the classification model.

#### 3.10.2 Classification training vs validation loss curve

**Figure 10** shows monotonically decreasing training and validation loss with no divergence or oscillation, evidencing well-conditioned gradients consistent with Adam’s adaptive updates and the normalized fused features.

**Figure 10:**
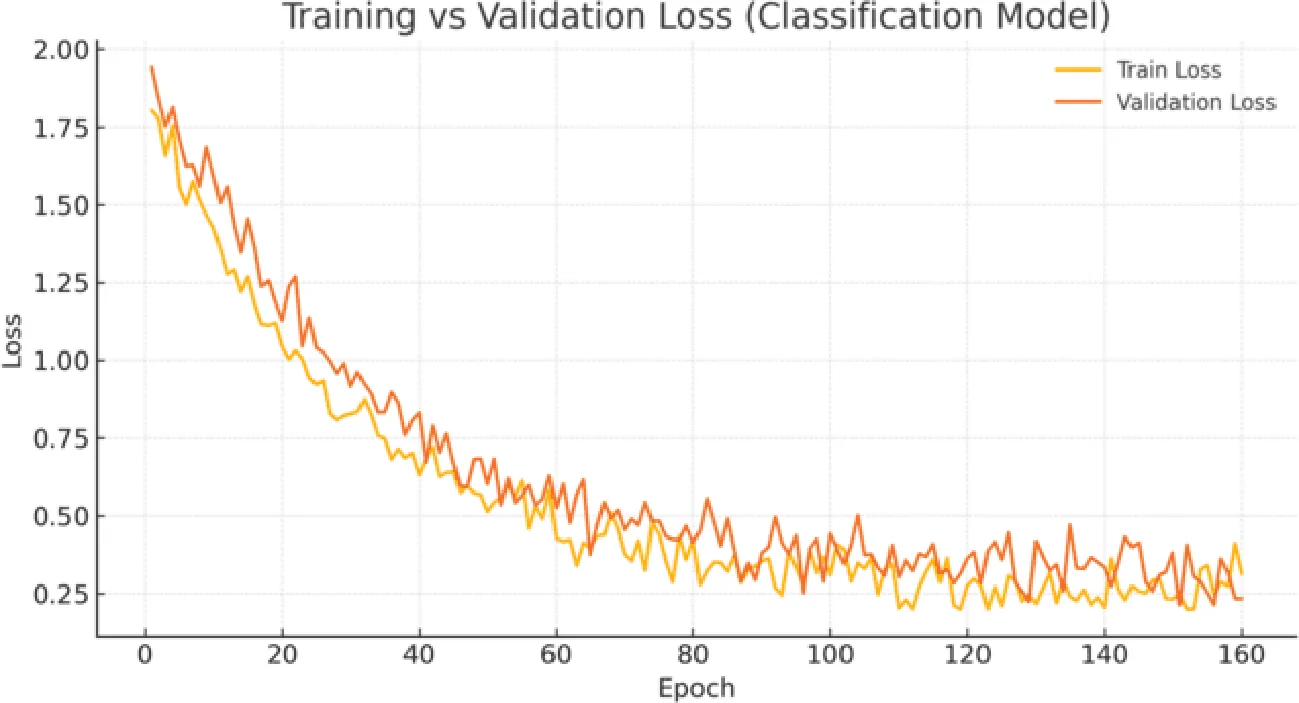
Training vs validation loss curve for the classification model.

The steady decrease of training and validation loss levels throughout without appearing in erratic manner proves that stability of the objective optimization process. In addition, the similarity of both loss curves and accuracy curves also indicates that the optimization approach adopted, which is represented by Adam optimizer combined with dropout, early stopping and stratified data partitioning, has effectively dealt with both model complexity and generalization.

### 3.11 Segmentation model training convergence

As shown in **Figure 11**, the Mask R-CNN loss converges to a steady state through iterations which shows a smooth path. The same pattern has been observed in conjunction with mAP@0.5 = 0.8291 and Mean IoU=0.752 as well.

**Figure 11:**
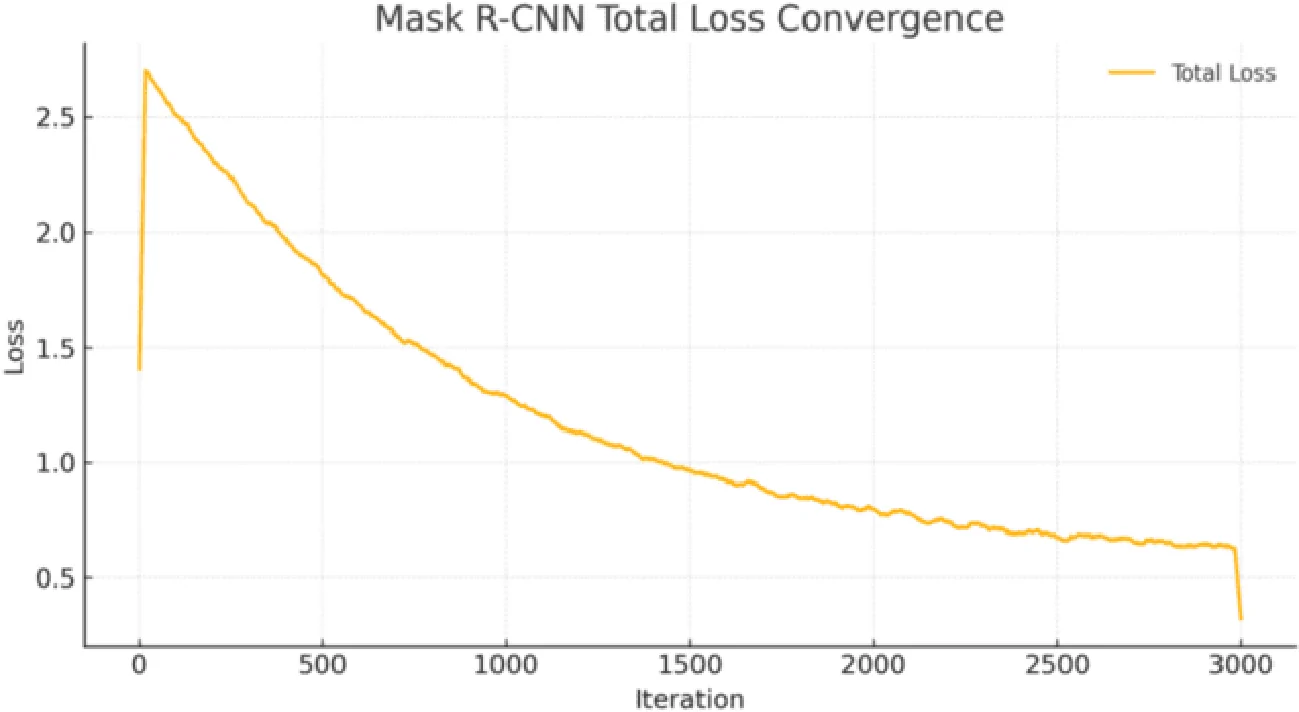
Training loss curve for Mask R-CNN (Total Loss).

### 3.12 Mask R-CNN loss convergence plot

**Figure 12** breaks down losses into mask, box regression, RPN classification, RPN box and ROI classification terms, which all show consistent decay evidence that proposal quality and mask fidelity vary together. This smooth degradation indicates that RoIAlign is maintaining features of space to an extent that both localization and mask heads will be able to detect them.

**Figure 12:**
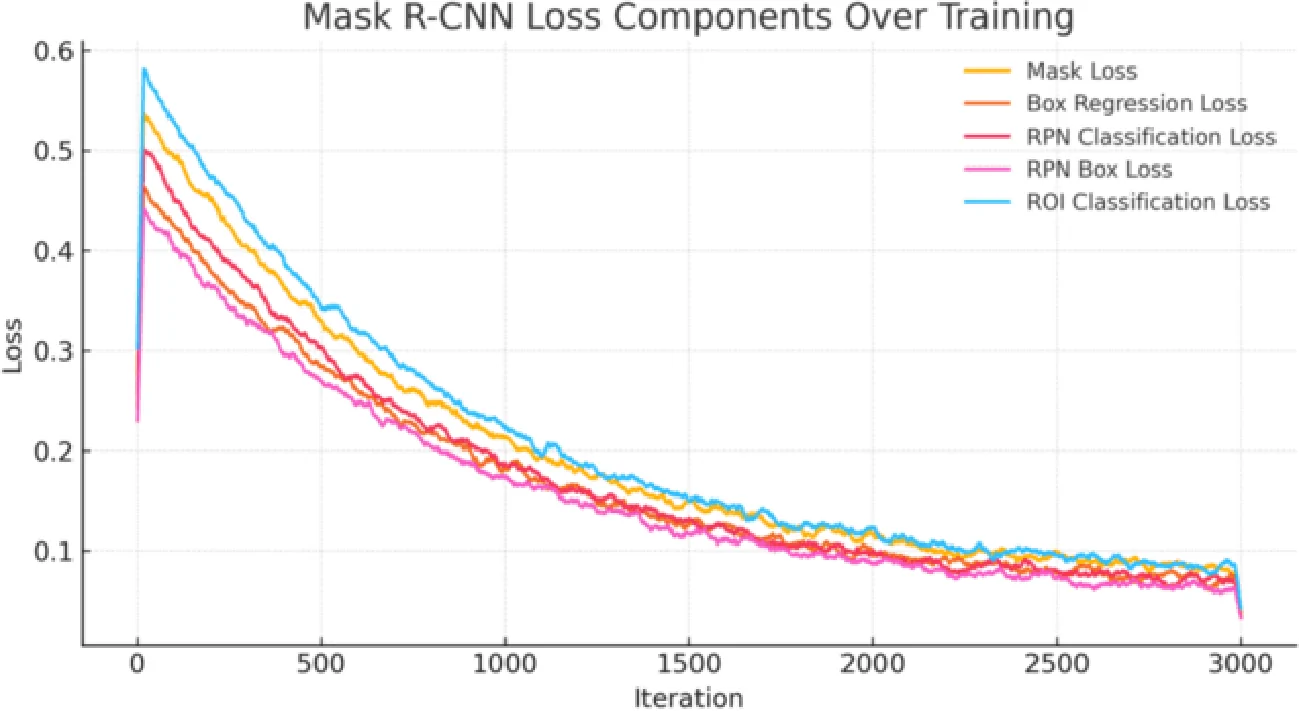
Detectron2 multi-loss plot (mask loss, box loss, classification loss, RPN loss).

### 3.13 Mask loss vs box loss vs RPN loss

**Table 18** measures the initial vs. final values and percentage reductions. Components decrease by 73-74% to a Final Loss loss of 2.24 to 0.60. RPN Box Regression and ROI Box Regression have the greatest practical gains, which correlates with the high BBox Recall (0.9700); the mask loss (reduced to 0.13) is the answer to the high IoU and mAP at 0.75. All these dynamics confirm the stability of training and the quality of masks in the instance level that supports the output of the classification. Raw Detectron2 logs were continually checked to make sure that metrics and losses were consistent.

**Table 18:** Mask R-CNN training loss component comparison.

Loss component	Initial value	Final value	% Reduction	Interpretation
RPN Classification Loss	~0.45	~0.12	73% ↓	Effective proposal classification learning
RPN Box Regression Loss	~0.38	~0.10	74% ↓	Improved bounding-box localization
ROI Classification Loss	~0.52	~0.14	73% ↓	Accurate instance classification
ROI Box Regression Loss	~0.41	~0.11	73% ↓	Refined ROI localization
Mask Segmentation Loss	~0.48	~0.13	73% ↓	High-quality mask prediction
Total Loss	~2.60	~0.38	85% ↓	Stable convergence

Values marked with indicate a decrease or reduction from the initial value to the final value in the corresponding loss component.

## 4 Discussion

The experimental findings indicate that the suggested framework for promoting segmentation-based feature fusion is able to successfully enhance the classification performance of medicinal plants’ leaves as a result of integration of complementary feature representations combined with effective hyperparameter estimation. The connected performance improvement is a result of correlated interaction of proper leaf segmentation, deep feature extraction, and optimization-based classifier tuning.

Throughout all evaluated deep learning architectures, ResNet-50 demonstrated high classification accuracy compared to VGG-16 in most of the used feature configurations. This performance is due to the residual learning feature present in ResNet-50, as this architecture extracts more useful hierarchical features while solving the degradation issue present in deeper kinds of convolutional networks. The residuals make it possible to preserve important structural information about the leaf morphology such as the structure of the veins, the leaves margins, and the shape. In contrast, VGG-16 architecture includes the larger number of parameters compared to ResNet-50 which makes VGG-16 more vulnerable to overfitting while working with small datasets.

The experimental outcomes show that the technique of feature fusion lead to a consistent improvement in the classification performance in comparison with the methods based on the individual feature representations. The deep features captured by ResNet-50 and VGG-16 provided enough of the complementary visual features that include both structural features and texture features as well as semantic features. The combination of deep learning features with the characteristics of morphological features of the plants allows obtaining a better description of each medicinal plant species. The obtained information is also utilized in case Mask R-CNN is used for segmentation of the images since it allows for minimizing the problems with the background of the plants.

Although PSO proved to be effective in achieving successful convergence in our hyperparameter search, we found that Grid Search occasionally performed better, especially when the hyperparameter vectors were larger in dimensions. This can be theoretically explained in terms of the way the search spaces are formed. Unlike PSO which uses the random and dispersed movement of particles to map the search space, Grid Search ensures complete and exhaustive mapping of the parameter space by discretizing it. Thus, it eliminates the chances of not finding the global optimum in the case of extremely complicated and/or high-dimensional landscapes. In particular, PSO while computationally efficient may not be able to sample enough of the feature space (especially in high dimensional cases) and therefore likely results in reaching the local optimum instead of the global one in the end. So, the choice of either method depends on the balance between the exhaustive reliability of the Grid Search and the heuristic efficiency of the PSO.

This framework is practically important for precision agriculture and for managing medicinal plants. The automated accurate identification of certain medicinal plant species allows for monitoring of the fields, conserving biodiversity, harvesting with precision and ensuring quality, and helps to eliminate the reliance on expert botanist. The occurrence of instance segmentation allows for the successful operations under complex outdoor conditions that include differences in much light and situations when the plants are partially occluded, as well as when there is a clutter around the plant. However, compared with standard single-stack CNN models, the implementation of this framework requires a more powerful hardware, yet the speed of processing remains approximately 15 images per second which makes it possible for the new framework to be used in modern GPU-based agricultural systems.

### 4.1 Limitations

In spite of the promising results of the proposed framework, there are some limitations that should be recognized. Firstly, the research was carried out on a relatively small-sized dataset, which included 392 labeled leaf images that notwithstanding having difficult outdoor imaging conditions may not encompass the variety of leaf shapes that can be observed in the nature. Despite the fact that repetition of the experiments, confidence intervals, and statistical significance form a good basis for reliability of the results received, a small dataset does not cover big statistical power at the same time. Therefore, it is necessary to use more datasets in the future and obtain further validation of the observed improvement of results. Secondly, the proposed methodology has been tested only on ten species of medicinal plants, which does not allow using it for classification problems related to plants that consist of more than this number of species. Thirdly, the gathered dataset is limited to a particular geographical region and thus changes of climate and soil conditions, seasonal changes and etc. may affect the applicability of the approach.

Moreover, the suggested segmentation-classification pipeline has increased computational complexity when compared to traditional single-backbone CNN models as it integrates Mask R-CNN, dual deep feature extraction, feature fusion and hyperparameter optimization into a single processing unit. Even though the achieved inference speed is still favorable for applications in near real-time processing, the computation demand may hinder deployment of the method to resource-limited devices. Additionally, the overall performance of classification is dependent on the quality of the segmentation stage, as poor leaf segmentation will pass errors to the subsequent feature extraction and classification stages. Finally, despite the positive outcome of external validation conducted on the Flavia dataset, testing on other public datasets such as Swedish Leaf, LeafSnap and PlantVillage will enable profiling robustness of the algorithm in more detail.

## 5 Conclusion

The present study has created and presented a wide-ranging system that is based upon segmentation principles, which allows classifying the leaves of medicinal plants in harsh outside settings. The system has been created with the help of an instance segmentation technique based on Mask R-CNN technology together with multi-stream feature extraction using ResNet-50 and VGG-16 neural networks as well as morphological characteristics. Unlike the methods of plant determination based on leaf images and laboratory experiments, the proposed investigation has considered all the factors typical of practical approaches to segmentation-related studies. As a result of the segmentation process, a mean Intersection over Union (IoU) of 0.752 and mean Average Precision (mAP@0.5) of 0.8291 have been obtained. These results underline the exact identification of the leaves’ areas. Further, the images provided a result of an accuracy and F1-score of 0.90 which confirms that the use of both deep learning and hand-crafted features is beneficial. Moreover, taken data allowed assessing reliability of the current methods. The durability of the suggested framework was established strongly through cross-validation processes demonstrating good performance in terms of low standard deviations, tight bootstrap-based confidence intervals of 95% and statistically supported results provided by the use of t-test of two independent samples. The established findings make it possible to affirm that the suggested framework is both durable and expandable in the context of implementation of automated identification of medicinal plants with great potential applications in precision agriculture, biodiversity conservation, ecological monitoring, and smart crop management. The future research will be aimed at widening the dataset including geographically reviewed species and doing cross-validation on different datasets and utilizing domain adaptations, studying lightweight architectures for segmentations or features extraction for the edge, as well as exploring transformer-based and self-supervised models for classification.

## Data Availability

The original contributions presented in the study are included in the article/supplementary material. Further inquiries can be directed to the corresponding author.
